# Piscine Vitamin D Receptors Vdra/Vdrb in the Absence of Vitamin D Are Utilized by Grass Carp Reovirus for Promoting Viral Replication

**DOI:** 10.1128/spectrum.01287-23

**Published:** 2023-07-19

**Authors:** Yun Jie Song, Jie Zhang, Jun Xiao, Hao Feng, Zhen Xu, Pin Nie, Ming Xian Chang

**Affiliations:** a State Key Laboratory of Freshwater Ecology and Biotechnology, Institute of Hydrobiology, Chinese Academy of Sciences, Wuhan, China; b College of Advanced Agricultural Sciences, University of Chinese Academy of Sciences, Beijing, China; c Innovation Academy for Seed Design, Chinese Academy of Sciences, Wuhan, China; d State Key Laboratory of Developmental Biology of Freshwater Fish, College of Life Science, Hunan Normal University, Changsha, China; Xinxiang Medical University

**Keywords:** RLR antiviral signaling pathway, vitamin D, cholesterol synthesis, RLR antiviral signaling, grass carp reovirus, viral inclusion bodies, vitamin D receptors

## Abstract

The vitamin D receptor (VDR) plays a pivotal role in the biological actions of vitamin D (VitD). However, little is known about the functions of VDR in the production of viral inclusion bodies (VIBs). Using a representative strain of grass carp reovirus (GCRV) genotype I, GCRV-873, we show that GCRV-873 recruits grass carp Vdrs for promoting the production of VIBs in the absence of VitD. Inhibition of cholesterol synthesis by lovastatin impairs the production of VIBs and blocks the effects of grass carp Vdrs in promoting the production of VIBs in the absence of VitD. Furthermore, grass carp Vdrs are found to form the Vdra-Vdrb heterodimer, which is vital for 3-hydroxy-3-methylglutaryl-coenzyme A reductase (*hmgcr*)-dependent cholesterol synthesis and GCRV replication. Intriguingly in the presence of VitD, grass carp Vdra but not Vdrb forms the heterodimer with the retinoid X receptor beta b (Rxrbb), which induces the transcription of those genes involved in the RIG-I-like receptor (RLR) antiviral signaling pathway for inhibiting GCRV infection. Furthermore, the VitD-activated Vdra-Vdrb heterodimer attenuates the transcription of the RLR antiviral signaling pathway induced by VitD. In the presence of VitD, a balance between the Vdra-Rxrbb heterodimers as coactivators and Vdra-Vdrb heterodimers as corepressors in affecting the transcriptional regulation of the RLR antiviral signaling pathway may eventually determine the outcome of GCRV infection. Transfection with VitD can abolish the effect of grass carp Vdrs in promoting GCRV replication in a dose-dependent manner. Taken together, these findings demonstrate that GCRV utilizes host Vdrs to increase *hmgcr*-dependent cholesterol synthesis for promoting its replication, which can be prevented by VitD treatment.

**IMPORTANCE** Grass carp reovirus (GCRV) is the causative agent of grass carp hemorrhagic disease, which seriously harms freshwater fish. Although many positive or negative regulators of GCRV infection have been identified in teleosts, little is known about the molecular mechanisms by which GCRV utilizes host factors to generate its infectious compartments beneficial for viral replication and infection. Here, we show that in the absence of VitD, the GCRV-873 strain utilizes host vitamin D receptors Vdra/Vdrb to increase *hmgcr*-dependent cholesterol synthesis for promoting the production of VIBs, which are important functional sites for aquareovirus replication and assembly. The negative regulation of Vdrs during viral infection can be prevented by VitD treatment. Thus, this present work broadens understanding of the pivotal roles of Vdrs in the interaction between the host and GCRV in the absence or presence of VitD, which might provide a rational basis for developing novel anti-GCRV strategies.

## INTRODUCTION

In mammals, bile acids have been shown to play complex physiological and pathological roles by activating a variety of receptors. There are two types of bile acid receptors, as follows: nuclear receptors and membrane receptors. Among these bile acid receptors, vitamin D receptor (VDR) is the only receptor that exists in two forms, namely, membrane VDR (mVDR) and nuclear VDR (nVDR) (1, 2). The ligand of VDR is vitamin D (VitD), a cholesterol derivative. In addition to regulating calcium and phosphorus metabolism, VitD also regulates the immune system, such as inducing the production of antimicrobial peptides to enhance antimicrobial activity against pathogens (3, 4), inducing autophagy (5), inhibiting adaptive immunity (6), and inhibiting the development of arthritis and recurrent encephalomyelitis (7, 8). However, VitD treatment might have a negative impact on the innate immune response elicited by viral infection and inflammatory responses of activated macrophages (9, 10).

The nuclear and membrane-bound VDRs render different functions after binding with VitD. The mVDR binding with VitD is involved in the activation of the second messenger system, rapid opening of Ca^2+^ channels, and adipocyte metabolism (2, 11, 12). The nVDR interacting with VitD heterodimerizes with retinoid X receptor (RXR) and binds target DNA sequences called vitamin D response elements (VDREs) to regulate the transcription of many pattern recognition receptors, cytokines/chemokines, antimicrobial peptides, and cathelicidin antimicrobial peptides, thereby ultimately affecting a variety of pathophysiological processes, including tumor, chronic kidney disease, cardiovascular disease, inflammation, and immune-related diseases (13–15). VDR can travel through the nuclear plasma independently of its ligand (16). Microtubule-associated protein 1 light chain 3 (LC3), a key regulator of autophagy, is found to interact with VDR and promote the nuclear translocation of VDR and the formation of the VDR-RXR heterodimer (17). The ligand-independent activity of VDR functions as a selective repressor or derepressor of gene expression and influences mammalian hair cycling (18, 19).

There are two paralogous genes of VDR in teleosts, namely, *vdra* and *vdrb*, which are generated by a whole-genome duplication event (20). Although most gene duplication events are nonfunctional and result in gene loss, in about 20 to 50% of cases, one copy may acquire a new beneficial function and be retained (21, 22). In response to VitD stimulation, the mVDRβ from medaka exhibits significant transactivation activity but not for mVDRα (20). In zebrafish, *vdrb* seems to be nonfunctional in Ca^2+^ homeostasis compared with *vdra*, and a loss of function of *vdra* was found to inhibit calcium uptake (21). For zebrafish heart and inner ear development, knock down of *vdra* has little effect, whereas disrupting the *vdrb* gene causes smaller otic vesicles with malformed semicircular canals, abnormal otoliths, pericardial edema, slower heart rate, and abnormal right jogging heart (23, 24). These studies suggest that the two paralogous *vdrs* may undergo functional divergence in teleosts. Vitamin D deficiency has been correlated with increased rates of infection, and the anti-infection activity of VitD has attracted extensive attention of researchers. Although, in mammals, activation of VDR by VitD administration inhibited Mycobacterium tuberculosis-induced bone destruction, and VDR^−/−^ mice exhibited significantly higher bacterial loading than wild-type VDR^+/+^ mice (25, 26). However, as yet, there have been no studies assessing the impact of paralogous genes of piscine *vdrs* in pathogen infection.

In this study, we demonstrated that viral infection induces the expression of *vdra* and *vdrb* in grass carp (Ctenopharyngodon idellus). VitD enhances innate antiviral immunity, whereas overexpression of grass carp *vdra* or *vdrb* impairs innate antiviral immunity. Furthermore, both grass carp Vdra and Vdrb interact with the nonstructural proteins NS38 and NS80 of grass carp reovirus (GCRV) and promote viral replication. Finally, our work elucidates the distinct mechanisms of grass carp Vdra/Vdrb in the regulation of GCRV infection in the presence or absence of VitD.

## RESULTS

### Sequence features of grass carp *vdra* and *vdrb*.

**FIG 1:**
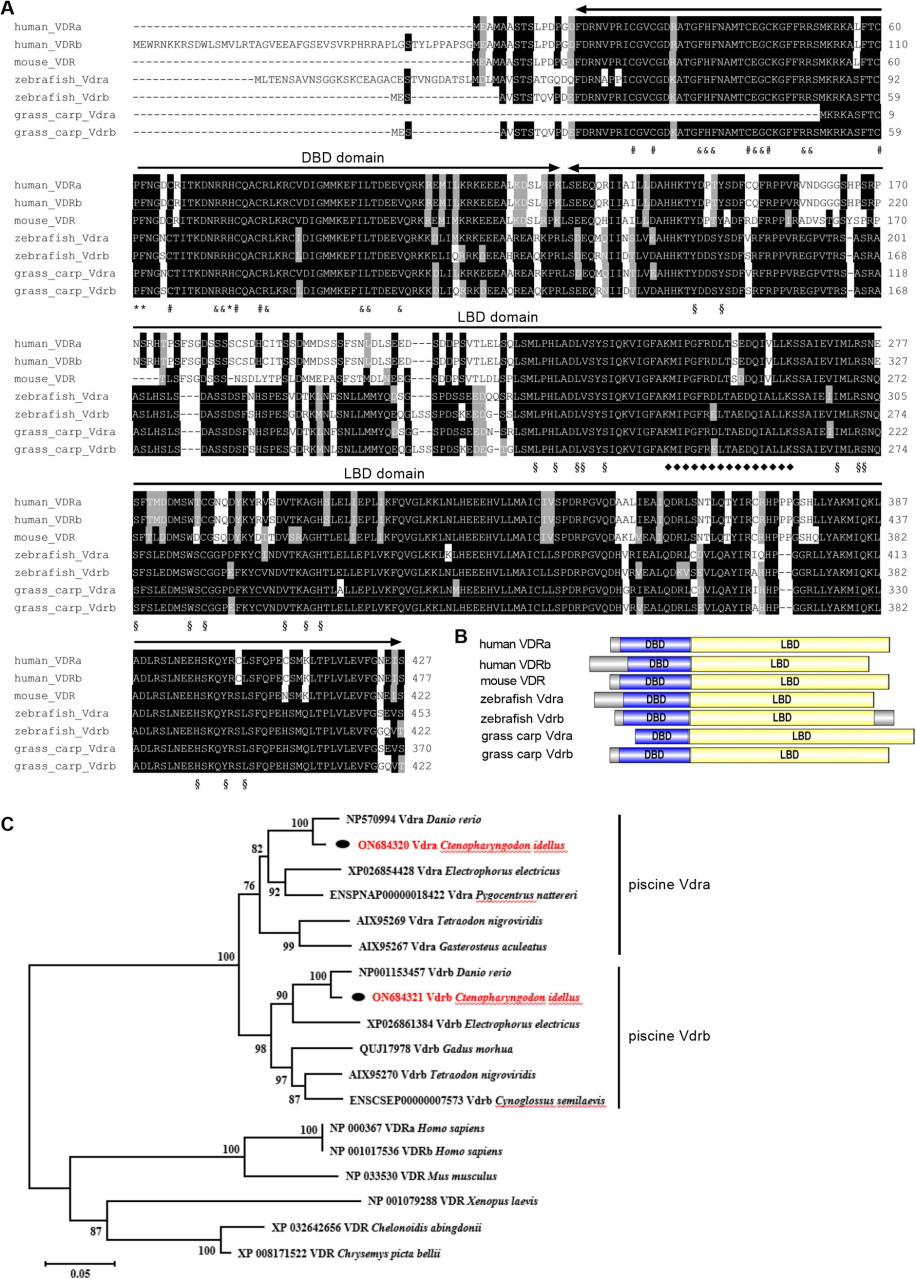
Sequence analysis of vertebrate VDRs. (A) Sequence alignments of vertebrate VDRs. #, indicates zinc binding site; δ, indicates DNA binding site; *, indicates dimer interface; §, indicates ligand binding site; ◆, indicates interaction with coactivator LXXLL motif. (B) The domains of vertebrate VDRs. (C) The evolutionary analysis of vertebrate VDRs. The grass carp Vdra and Vdrb are highlighted in red. DBD, DNA-binding domain; LBD, ligand binding domain.

In grass carp, we cloned and obtained 2 *vdr*s. The whole open reading frame (ORF) of grass carp *vdra* (GenBank accession number ON684320) encodes 370 amino acids, which shows 71% identity to human VDRa and VDRb, 97.3% identity to zebrafish Vdra, and 89.5% identity to zebrafish Vdrb. The whole ORF of grass carp *vdrb* (GenBank accession number ON684321) encodes 422 amino acids, which shows 71.1% identity to human VDRa and VDRb, 88.1% identity to zebrafish Vdra, 97.9% identity to zebrafish Vdrb, and 89.2% identity to grass carp Vdra. A sequence analysis of grass carp Vdra and Vdrb revealed the existence of DNA-binding domain (DBD) and ligand-binding domain (LBD) (Fig. 1A and B).

Next, a phylogenetic analysis with full-length Vdr sequences was conducted in order to compare phylogenetic relationships of Vdra and Vdrb throughout vertebrate evolution. Human VDRa and VDRb clustered with the VDRs from the other mammalian VDR, amphibian VDR, and reptile VDRs. The piscine Vdra and Vdrb form two separate subclusters (Fig. 1C).

### Expression and subcellular localization of grass carp *vdra* and *vdrb*.

**FIG 2:**
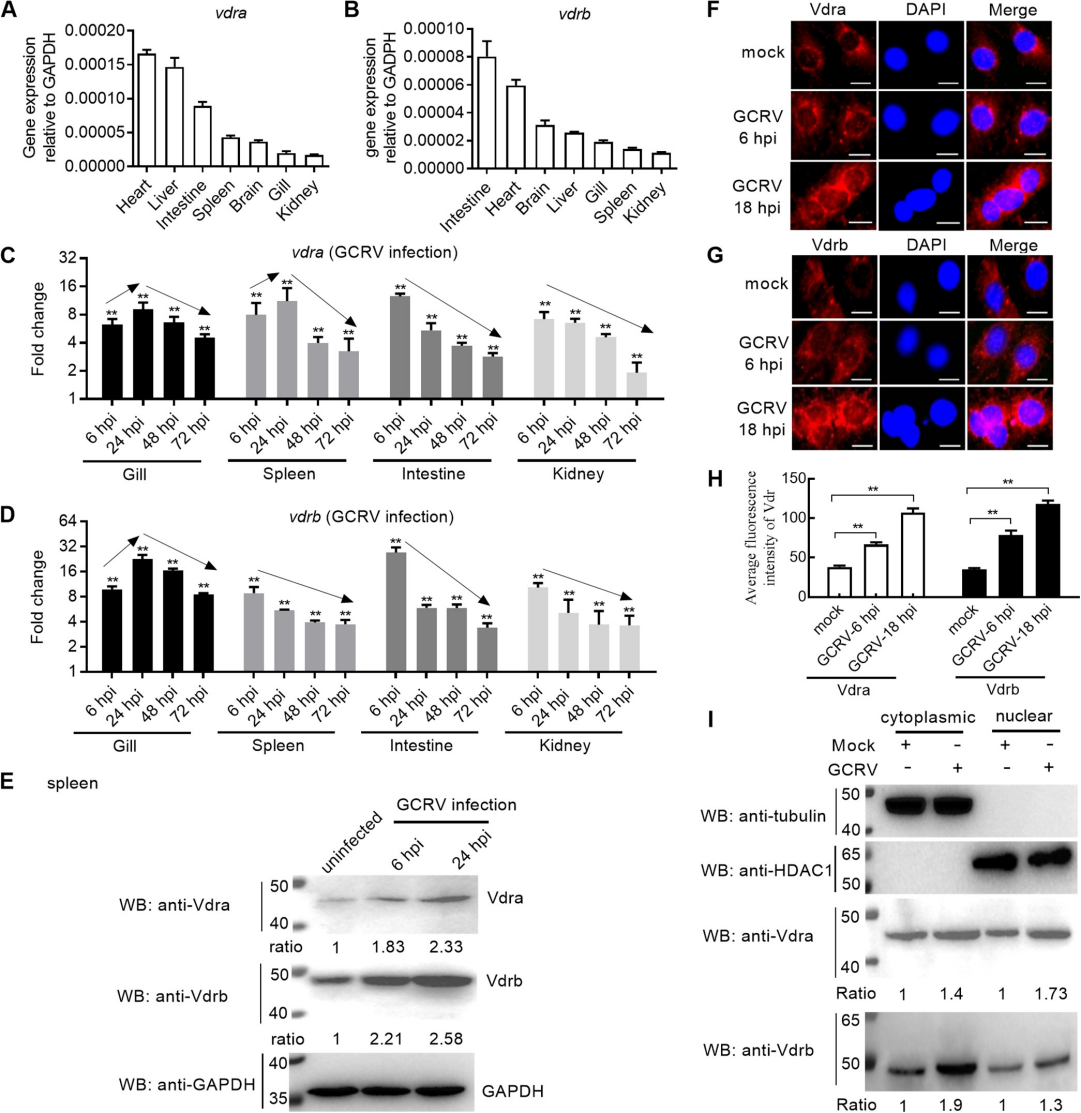
The expressions and subcellular localizations of grass carp *vdra* and *vdrb*. (A and B) The constitutive expression of grass carp *vdra* and *vdrb* in different tissues. The liver, brain, skin, intestine, gill, kidney, and spleen from three healthy fish were collected and used for qRT-PCR. (C and D) The inducible expression of grass carp *vdra* or *vdrb* in gill, spleen, intestine, and kidney in response to GCRV infection. The gill, spleen, intestine, and kidney from 3 mock-infected and 3 GCRV-infected grass carp were collected at 6, 24, 48, and 72 hpi and used for qRT-PCR. Arrows indicate the trend of induced expression of grass carp *vdra* or *vdrb.* The asterisks above the error bars indicated statistical significance using the group without GCRV infection as the control group. (E) The protein expressions of grass carp Vdra and Vdrb in the spleen from the uninfected and GCRV-infected grass carp. The spleen from GCRV-infected grass carp was collected at 6 and 24 hpi. Protein bands were quantified by Image J. (F and G) The subcellular localizations of grass carp Vdra or Vdrb in CIK cells with or without the GCRV infection. Scale bars, 10 μm. (H) The average fluorescence intensity of grass carp Vdra or Vdrb in CIK cells with or without the GCRV infection. The asterisks above the bracket indicated statistical significance between the two groups connected by the bracket. For F to H, CIK cells plated onto coverslips in 24-well plates were infected with GCRV at an MOI of 1 or left untreated. At 6 and 18 hpi, the cells were washed and fixed with 4% PFA for immunofluorescence assays. DAPI, 4′,6-diamidino-2-phenylindole. The Image J was used to detect the mean fluorescence intensity. (I) The cytoplasmic and nuclear expressions of grass carp Vdra and Vdrb in CIK cells with or without the GCRV infection. The mock-infected cells and the cells infected with GCRV at an MOI of 1 for 24 h were collected and used for protein extraction. Protein bands were quantified by Image J. Data are presented as mean values ± SD (*n* = 3), and *P* values by Student’s *t* test are shown in A to D and H. **, *P < *0.01.

In order to clarify the target organ(s) of piscine *vdr*s, the constitutive expressions of grass carp *vdra* and *vdrb* in different tissues were first examined. The expression of grass carp *vdra* is the highest in heart and then in the liver and intestine (Fig. 2A). Different from grass carp *vdra*, the expression of grass carp *vdrb* is the highest in intestine, followed by heart and brain (Fig. 2B). The expression of both *vdra* and *vdrb* is the lowest in kidney (Fig. 2A and B).

To clarify the possible immune regulation of grass carp on *vdr* in the presence of GCRV infection, the lymphoid (spleen and kidney) and mucosal (gill and intestine) immune tissues are used to investigate the inducible expression levels of grass carp *vdra* and *vdrb*. The mRNA levels of grass carp *vdra* and *vdrb* were induced by GCRV infection in all tested immune tissues (Fig. 2C and D). In the gill and spleen, the induced expression of grass carp *vdra* was the highest at 24 h postinfection (hpi) and decreased thereafter. In the intestine and kidney, the induced expression of grass carp *vdra* was the highest at 6 hpi and decreased thereafter (Fig. 2C). In the gill, the induced expression of grass carp *vdrb* was the highest at 24 hpi and decreased thereafter. In the spleen, intestine, and kidney, the induced expression of grass carp *vdrb* was the highest at 6 hpi and decreased thereafter (Fig. 2D).

Next, the protein levels of grass carp Vdra and Vdrb were examined using the polyclonal anti-Vdra and anti-Vdrb antibodies. Antibody (Ab) specificity was first verified by immunoblotting. The polyclonal anti-Vdra or anti-Vdrb antibody can detect a strong single endogenous Vdra or Vdrb protein band, whose molecular weight is consistent with that of the exogenous Vdra-FLAG (see Fig. S1A in the supplemental material) or Vdrb-FLAG (Fig. S1B). The abovementioned results from reverse transcription-quantitative PCR (qRT-PCR) have shown that the induced expression trends of grass carp *vdra* and *vdrb* from 6 to 72 hpi were similar in gill, intestine, and kidney; however, they were somewhat different in the spleen. Moreover, the difference between the induced expression trends of grass carp *vdra* and *vdrb* was observed from 6 to 24 hpi, with the highest increase in the expression of grass carp *vdra* at 24 hpi but that at 6 hpi for grass carp *vdrb*. Based on these data, we further examined the protein expressions of Vdra and Vdrb in the spleen of grass carp collected at 6 and 24 hpi after confirming the specificity of grass carp Vdra and Vdrb antibodies. The results of Western blotting demonstrated that GCRV infection significantly induced the endogenous expressions of grass carp Vdra and Vdrb at 6 and 24 hpi (Fig. 2E). However, the increase in the expression of grass carp Vdra and Vdrb was the higher at 24 hpi than that at 6 hpi.

To investigate whether the subcellular location of piscine Vdr is affected by virus infection, the endogenous subcellular localizations of grass carp Vdra and Vdrb are examined using anti-Vdra or anti-Vdrb antibody in the mock-infected or GCRV-infected *C. idellus* kidney (CIK) cells. In the mock-infected CIK cells, grass carp Vdra and Vdrb were found to exist mainly in the cytoplasm and weakly in the nucleus. In the GCRV-infected CIK cells, the subcellular localizations of grass carp Vdra and Vdrb were rather obvious both in the cytoplasm and nucleus, especially at 18 hpi (Fig. 2F and G). GCRV infection significantly enhanced the intracellular accumulations of grass carp Vdra and Vdrb (Fig. 2H). Compared with the mock-infected control group, GCRV infection increased nuclear accumulations of grass carp Vdra and Vdrb with a 1.73-fold and 1.30-fold increase, respectively, and 1.40-fold and 1.90-fold increase, respectively, for the cytoplasmic accumulations of grass carp Vdra and Vdrb (Fig. 2I).

These results suggest that GCRV infection induces the expression levels of grass carp Vdra and Vdrb both in the cytoplasm and nucleus.

### Grass carp *vdra* and *vdrb* in the absence of VitD promote GCRV replication, and *vdr*-mediated GCRV replication can be blocked by VitD.

**FIG 3:**
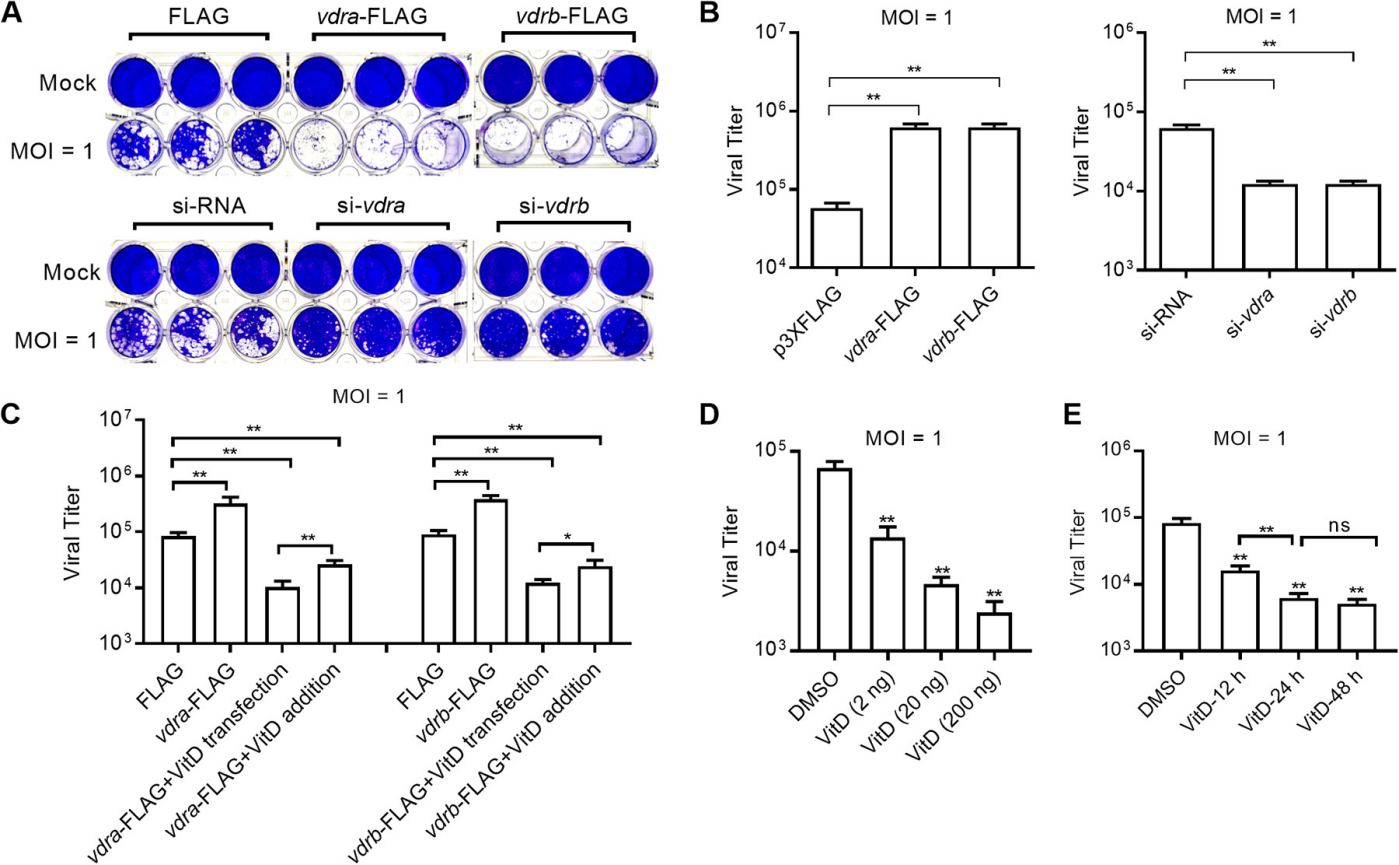
The role of grass carp *vdrs* and VitD in GCRV infection. (A and B) Crystal violet staining and determination of GCRV titers for overexpression or knockdown of grass carp *vdra* or *vdrb*. CIK cells grown in 24-well plates were transfected with 800 ng FLAG empty plasmid, *vdra*-FLAG, or *vdrb*-FLAG; or 100 nM siRNA, si-*vdra*, or si-*vdrb*. (C) Determination of GCRV titers for overexpression of grass carp *vdra* or *vdrb* in CIK cells with or without the treatment of VitD. CIK cells grown in 24-well plates were transfected with 800 ng FLAG, *vdra*-FLAG, or *vdrb*-FLAG, altogether with the transfection of 20 ng VitD for 24 h or the addition of 200 ng VitD to the medium for 24 h or left untreated. (D) Determination of GCRV titers for transfection of various concentrations of VitD. CIK cells grown in 24-well plates were transfected with 2 ng VitD, 20 ng VitD, 200 ng VitD, or DMSO. (E) Determination of GCRV titers for transfection of VitD for various timing before these cells were infected by GCRV. CIK cells grown in 24-well plates were transfected with 20 ng VitD. After 12, 24, and 48 h posttransfection, the cells were infected with GCRV. For B to E, these cells were infected with GCRV at an MOI of 1 for 24 h and then collected for determining GCRV titers. Data are presented as mean values ± SD (*n* = 3). For B, C, and E, the asterisks above the bracket indicated statistical significance between the two groups connected by the bracket. For D and E, the asterisks above the error bars indicated statistical significance using the group transfected with DMSO as the control group. **, *P < *0.01; ns, not significant.

The roles of mammalian VDR or VitD analogs during different viral infections are controversial. We are interested to know how piscine *vdr* and VitD affect the replication and infection of GCRV. To further investigate the function of grass carp *vdr*, three small interfering RNAs (siRNAs) targeting the mRNAs of grass carp *vdra* or *vdrb* were synthesized. The silencing efficiencies of these siRNAs were determined by qRT-PCR. A preliminary experiment indicated that si-*vdra*2 and si-*vdrb*2 possessed the best silencing efficiency at a final concentration of 100 nM, which were used for the follow-up research (Fig. S1C and D). We found that in the absence of VitD, overexpression of grass carp *vdra* or *vdrb* increased the sensitivity of CIK cells to GCRV infection, and si-*vdr*-mediated knockdown of grass carp *vdra* or *vdrb* was much more resistant to GCRV infection than the cells transfected with siRNA (Fig. 3A). Furthermore, the higher viral load was observed in the CIK cells transfected with grass carp *vdra* or *vdrb* than that in the control group transfected with FLAG empty plasmid. Compared with the cells transfected with siRNA, the viral load was lower in the CIK cells transfected with si-*vdra* or si-*vdrb* (Fig. 3B).

In the presence of VitD, overexpression of grass carp *vdra* or *vdrb* significantly inhibited GCRV replication compared with the control group transfected with FLAG (Fig. 3C). When studying the function of the intracellular receptor *in vitro*, transfection of ligand is more effective than adding ligand to the medium. Compared with the group with the transfection of grass carp *vdr* and the VitD addition to the medium, the lower viral load was observed in the CIK cells transfected with both grass carp *vdr* and VitD (Fig. 3C). Therefore, we use transfection of VitD rather than adding VitD to the medium for the follow-up research.

To investigate the role of VitD in GCRV infection, dose-response assays and time-lapse studies were used to investigate the functions of VitD. Increasing concentrations of VitD transfection from 2 ng to 200 ng per well in 24-well plate inhibited GCRV replication in a dose-dependent manner (Fig. 3D). Transfection of VitD for 12, 24, and 48 h before these cells were infected by GCRV inhibited GCRV replication. A continuous decrease of GCRV titers was seen from 12 h to 24 h transfection of VitD before these cells were infected by GCRV, and there was no further change from 24 h to 48 h transfection of VitD before these cells were infected by GCRV (Fig. 3E).

### Grass carp *vdra* and *vdrb* in the absence of VitD are utilized by GCRV for promoting the expressions of GCRV proteins.

**FIG 4:**
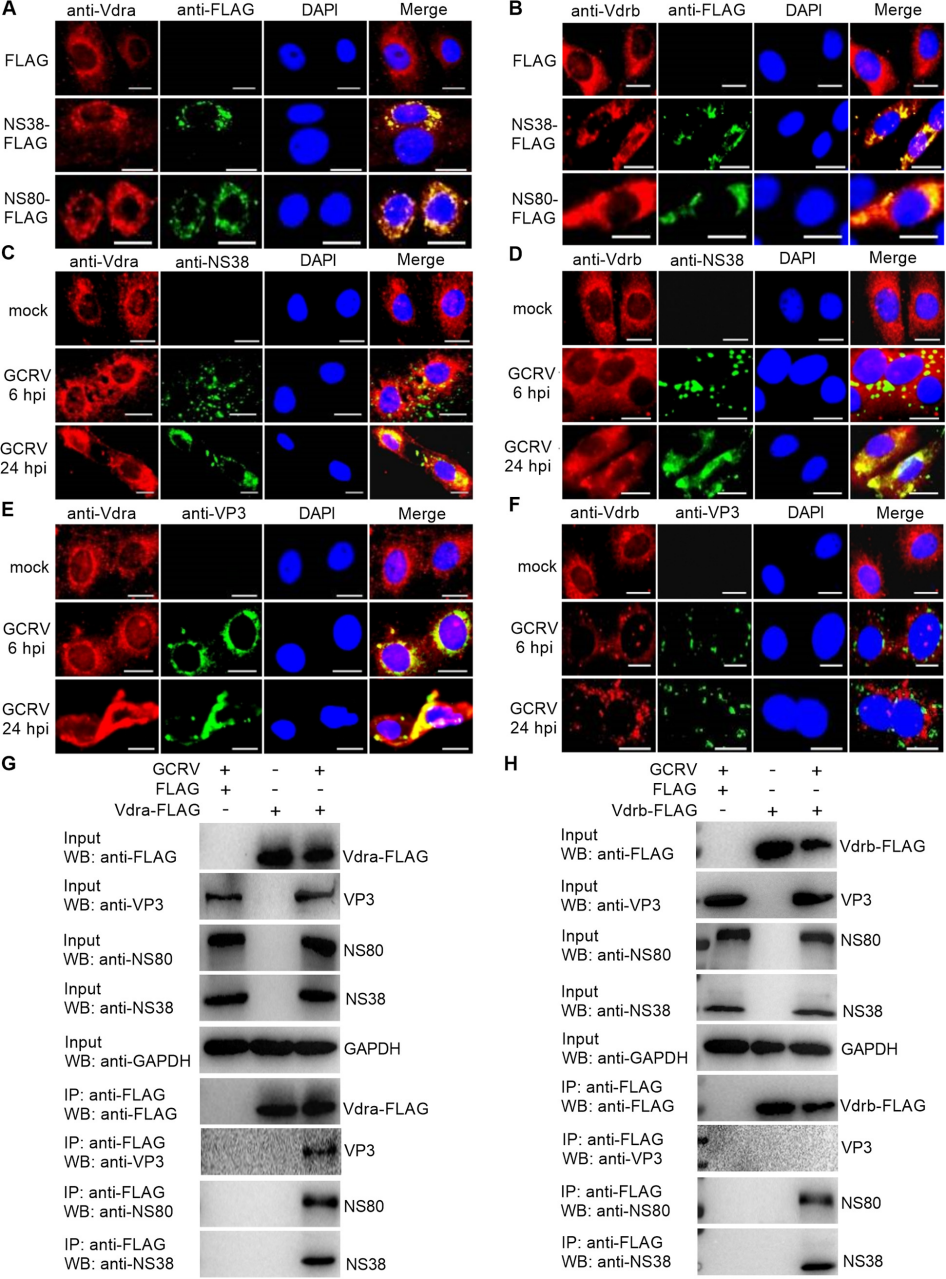
The subcellular colocalizations or interactions between grass carp Vdrs and GCRV proteins. (A and B) The subcellular colocalizations between grass carp Vdra or Vdrb and the exogenous NS38 or NS80 of GCRV. CIK cells plated onto coverslips in 24-well plates were transfected with 800 ng NS38-FLAG or NS80-FLAG. After 24 h posttransfection, the cells were washed and fixed with 4% PFA for immunofluorescence assays. (C to F) The subcellular colocalizations between the endogenous Vdra or Vdrb and NS38 or VP3 of GCRV. CIK cells plated onto coverslips in 24-well plates were infected with GCRV at an MOI of 1 or left untreated. At 6 and 24 hpi, the cells were washed twice with PBS and fixed with 4% PFA for immunofluorescence assays. For A to F, the images were obtained using a SP8 Leica laser confocal microscopy imaging system. Scale bars, 10 μm. (G and H) The interactions between grass carp Vdra or Vdrb and GCRV proteins. CIK cells seeded in 10-cm^2^ dishes were transfected with 10 μg FLAG, *vdra*-FLAG, or *vdrb*-FLAG. After 24 h posttransfection, the cells were infected with GCRV at an MOI of 1 or left untreated. At 24 hpi, the cells were harvested and used for co-IP.

Since the replication and assembly of GCRV take place in VIBs and the nonstructural proteins NS38 and NS80 of GCRV are viral proteins that form VIBs, we thought it would be interesting to know whether grass carp *vdr* is targeted by NS38 and NS80 of GCRV. We first investigated the possible interactions between grass carp Vdra or Vdrb and NS38 or NS80 of GCRV. When CIK cells were transfected with NS38-FLAG or NS80-FLAG, the endogenous grass carp Vdra or Vdrb colocalized with the cytoplasmic NS38 and NS80 (Fig. 4A and B). When CIK cells were infected with GCRV, the endogenous grass carp Vdra or Vdrb was also found to colocalize with the cytoplasmic NS38 (Fig. 4C and D). The endogenous grass carp Vdra also colocalized with the cytoplasmic VP3 structural protein of GCRV but not for grass carp Vdrb (Fig. 4E and F). To test whether grass carp Vdra and Vdrb interacted with GCRV proteins, CIK cells were transfected with FLAG-tagged Vdra or Vdrb and then infected with GCRV or left untreated. The interactions between FLAG and NS80/NS38/VP3 were examined as the negative controls. Both grass carp Vdra and Vdrb were associated with NS38 and NS80 of GCRV. However, the endogenous grass carp Vdra could interact with the VP3 of GCRV, and grass carp Vdrb failed to interact with the VP3 of GCRV (Fig. 4G and H).

**FIG 5:**
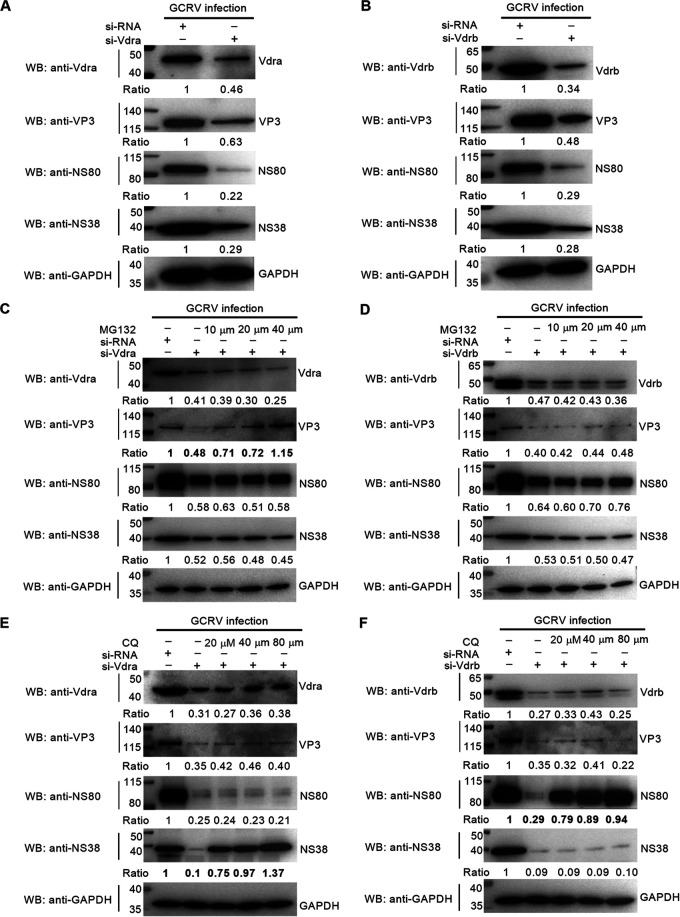
The effect of grass carp Vdra and Vdrb on the expressions of GCRV proteins. (A and B) The effect of grass carp Vdra or Vdrb knockdown on the expressions of GCRV proteins. CIK cells grown in 6-well plates were transfected with 100 nM siRNA, si-Vdra, or si-Vdrb. After 24 h posttransfection, the cells were infected with GCRV at an MOI of 1 for 24 h and then used for Western blotting. (C to F) The effect of MG132 or CQ on the degradation of GCRV proteins mediated by knockdown of grass carp Vdra or Vdrb. CIK cells seeded in 6-well plates overnight were transfected with 100 nM siRNA, si-Vdra, or si-Vdrb. After 24 h transfection, the cells were treated with MG132 or CQ at the indicated concentration for another 6 h or left untreated. Then, these cells were collected and used for Western blotting. For A to F, protein bands were quantified by Image J.

We next determined whether grass carp Vdra and Vdrb could promote the expressions of GCRV proteins. As shown in Fig. 5A and B, knockdown of grass carp Vdra or Vdrb resulted in the decrease in the expression levels of NS38, NS80, and VP3 proteins. We further investigate the effects of MG132 (inhibitor for the proteasome), 3-methyladenine (3-MA; early autophagy inhibitor), and chloroquine (CQ; late autophagy inhibitor) on the Vdra or Vdrb knockdown-mediated suppressions of GCRV proteins. Immunoblot analysis showed that the abundance of VP3 impaired by Vdra knockdown was restored by MG132 but not for NS38 and NS80 proteins (Fig. 5C). The abundance of VP3, NS38, and NS80 impaired by Vdrb knockdown was not restored by MG132 (Fig. 5D). 3-MA failed to restore the protein degradation of VP3, NS38, and NS80, which were mediated by knockdown of grass carp Vdra or Vdrb (Fig. S1E and F). CQ could restore the protein levels of NS38 impaired by Vdra knockdown and NS80 impaired by Vdrb knockdown (Fig. 5E and F).

Taken together, these results suggest that grass carp *vdra* and *vdrb* in the absence of VitD are utilized by GCRV for promoting the expressions of GCRV proteins.

### Grass carp *vdra* and *vdrb* in the absence of VitD promote the production of VIBs during GCRV infection, which can be blocked by lovastatin, a potent inhibitor of cholesterol biosynthesis.

**FIG 6:**
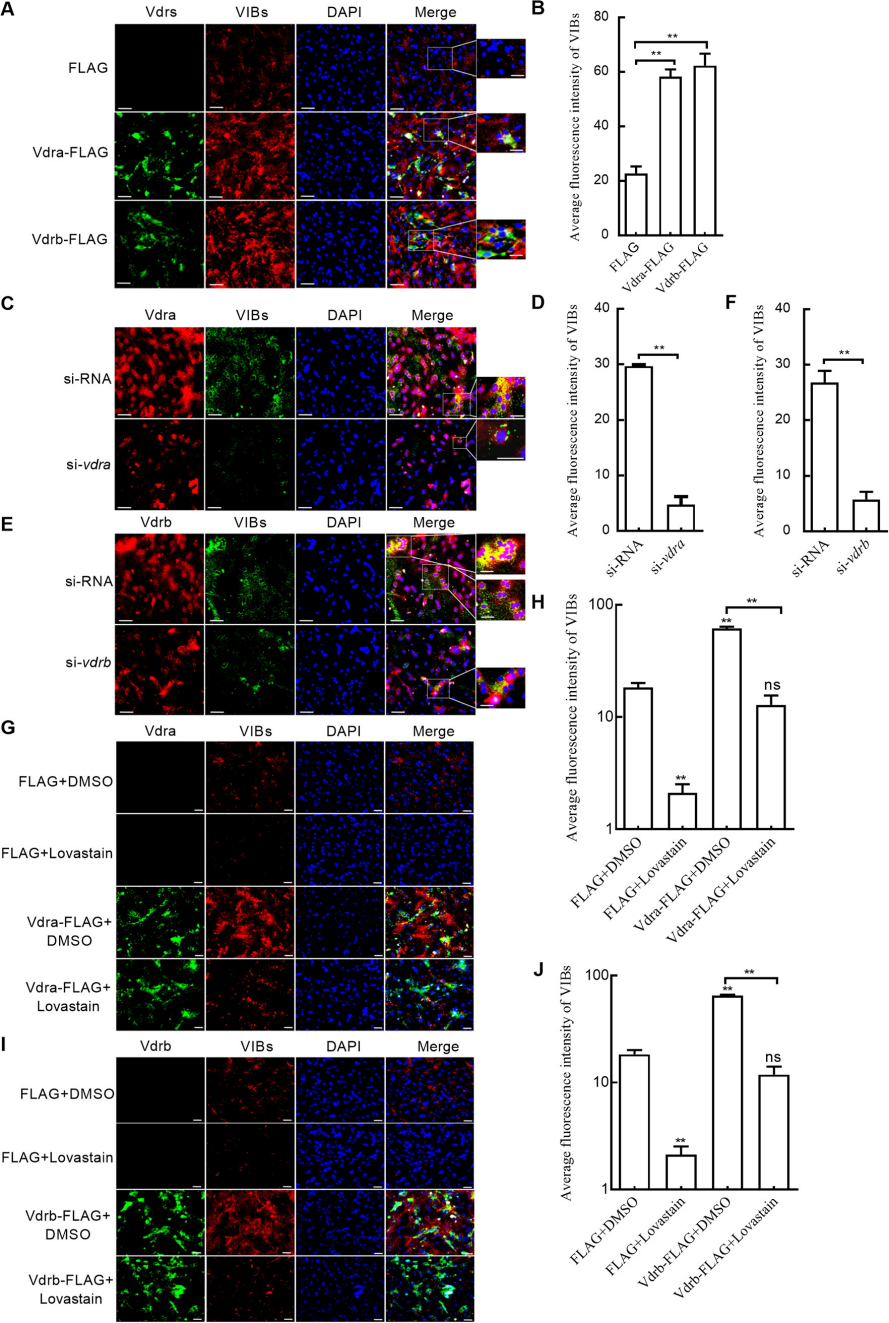
The effect of grass carp Vdrs in the absence of VitD on the production of VIBs. (A) The effect of overexpression of grass carp Vdra or Vdrb on the production of VIBs. Scale bars, 50 μm. (B) The average fluorescence intensity of VIBs in CIK cells overexpressed with grass carp Vdra or Vdrb. (C) The effect of knockdown of grass carp Vdra on the production of VIBs. Scale bars, 50 μm. (D) The effect of knockdown of grass carp Vdra on the average fluorescence intensity of VIBs. (E) The effect of knockdown of grass carp Vdrb on the production of VIBs. Scale bars, 50 μm. (F) The effect of knockdown of grass carp Vdrb on the average fluorescence intensity of VIBs. (G and H) The immunofluorescence images and average fluorescence intensity of VIBs regulated by lovastatin or/and grass carp Vdra. Scale bars, 50 μm. (I and J) The immunofluorescence images and average fluorescence intensity of VIBs regulated by lovastatin or/and grass carp Vdrb. Scale bars, 50 μm. For A to F, CIK cells plated onto coverslips in 24-well plates were transfected with 800 ng FLAG, *vdra*-FLAG, or *vdrb*-FLAG or with 100 nM siRNA, si-*vdra*, or si-*vdrb*. After 24 h, the cells were infected with GCRV at an MOI of 1. At 24 hpi, the cells were collected and then fixed with 4% PFA for immunofluorescence assays. Image J was used to detect the mean fluorescence intensity. For G to J, CIK cells plated onto coverslips in 24-well plates were transfected with 800 ng FLAG, *vdra*-FLAG, or *vdrb*-FLAG. After 24 h, the cells were infected with GCRV at an MOI of 1. At 18 hpi, the infected cells were incubated with 80 μM lovastatin for 6 h or left untreated. Then, the cells were washed and fixed with 4% PFA for immunofluorescence assays. Image J was used to detect the mean fluorescence intensity. For H and J, the asterisks above the error bars indicated statistical significance using the group transfected with DMSO and FLAG plasmid as the control group. For B, D, F, H, and J, the asterisks above the bracket indicated statistical significance between the two groups connected by the bracket. **, *P < *0.01; ns, not significant.

Since grass carp Vdra and Vdrb could promote the expressions of NS80 and NS38 proteins, whether grass carp Vdrs were beneficial for the production of VIBs was further investigated. Using the anti-NS80 antibody, the results of immunofluorescence analysis showed that overexpression of grass carp Vdra or Vdrb significantly increased the total amounts of VIBs compared with the control group transfected with FLAG (Fig. 6A and B). Compared with the control group transfected with siRNA, knockdown of grass carp *vdra* or *vdrb* enormously impaired the production of VIBs (Fig. 6C to F).

Previous study has shown that cholesterol is required for GCRV entry. Here, we were interested to know whether cholesterol biosynthesis affects the production of VIBs. Lovastatin is an inhibitor of cholesterol biosynthesis. In GCRV-infected cells, the total amounts of VIBs were decreased by lovastatin treatment (Fig. 6G to J). Furthermore, lovastatin treatment completely blocked the production of VIBs induced by grass carp Vdra or Vdrb (Fig. 6G to J).

All these data suggest that grass carp *vdra* and *vdrb* in the absence of VitD promote the production of VIBs, which is dependent on cholesterol biosynthesis.

### Grass carp Vdra and Vdrb in the absence of VitD fail to form the heterodimer with Rxr but form a heterodimer with each other for promoting the transcription of genes involved in cholesterol biosynthesis.

**FIG 7:**
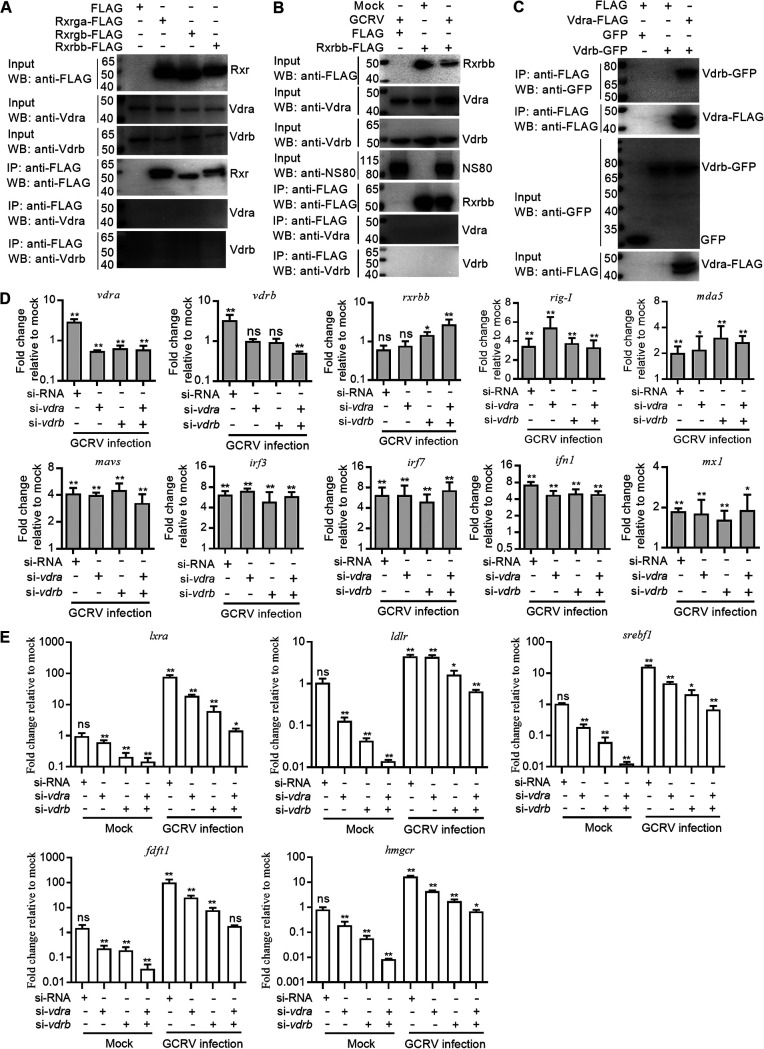
Grass carp *vdra* and *vdrb* in the absence of VitD promote GCRV replication via a manner independent of the RLR antiviral signaling pathway but dependent of cholesterol biosynthesis. (A) The interaction between Vdr and Rxr in the absence of VitD. CIK cells seeded in 10-cm^2^ dishes were transfected with 10 μg FLAG, *rxrga*-FLAG, *rxrgb*-FLAG, or *rxrbb*-FLAG. After 48 h posttransfection, the cells were harvested and used for Co-IP. (B) GCRV infection failed to trigger the interaction between grass carp Vdra or Vdrb and Rxrbb. CIK cells seeded in 10-cm^2^ dishes were transfected with 10 μg FLAG or *rxrbb*-FLAG. After 24 h posttransfection, the cells were infected with GCRV at an MOI of 1 or left untreated. At 24 hpi, the cells were harvested and used for co-IP. (C) The interaction between grass carp Vdra and Vdrb. CIK cells seeded in 10-cm^2^ dishes were transfected with 10 μg FLAG, *vdra*-FLAG, GFP, or *vdrb*-GFP. After 48 h posttransfection, the cells were harvested and used for co-IP. (D) The effects of knockdown of grass carp *vdra* or *vdrb* on the GCRV-activated expressions of many genes involved in the RLR antiviral signaling pathway. (E) The effects of knockdown of grass carp *vdra* or/and *vdrb* on the transcriptional levels of many genes involved in cholesterol biosynthesis. For D and E, CIK cells grown in 6-well plates were transfected with 100 nM siRNA, si-*vdra*, or/and si-*vdrb*. After 24 h posttransfection, these cells were infected with GCRV at an MOI of 1 or left untreated. At 24 hpi, these cells were collected and used for qRT-PCR. Data are presented as mean values ± SD (*n* = 3). The asterisks above the error bars indicated statistical significance using the group transfected with siRNA without GCRV infection as the control group. *, *P < *0.05; **, *P < *0.01; ns, not significant.

Binding of VitD to the full-length VDR causes destabilization of the VDR homodimer and formation of a heterodimeric complex with the RXR (27). In vertebrates, there are typically three copies of RXR genes, which include RXRα, RXRβ, and RXRγ. In zebrafish, additional copies of *rxra*, *rxrb*, and *rxrg* are found (28). Here, we obtained only the orthologous genes of *rxrbb* (GenBank accession number ON684322), *rxrga* (GenBank accession number ON720278), and *rxrgb* (GenBank accession number ON720279) from grass carp. To determine the possible interaction between piscine Vdrs and Rxrs, CIK cells were transfected with FLAG-tagged Rxrbb, Rxrga, Rxrgb, or the corresponding control and then infected with GCRV or left untreated. In the absence of VitD, neither grass carp Vdra nor Vdrb interacted with the Rxrga, Rxrgb, or Rxrbb without or with the GCRV infection (Fig. 7A and B). Interestingly, grass carp Vdra and Vdrb could form a heterodimer with each other in the absence of VitD (Fig. 7C).

VDR is a rare nuclear receptor that can play a regulatory role without ligand binding and can form homologous dimers (VDR-VDR) to regulate gene expression. Since grass carp Vdra and Vdrb can form a Vdra-Vdrb complex independent of VitD, we next investigated whether grass carp *vdra* and *vdrb* promoted GCRV replication and infection via impairing the classical RLR antiviral signaling pathway in the absence of VitD. Unexpectedly, knockdown of grass carp *vdra* or *vdrb* and even double knockdown of grass carp *vdra* and *vdrb* had no marked effects on the GCRV-activated expressions of many genes involved in the RLR antiviral signaling pathway (Fig. 7D).

Since lowering cholesterol biosynthesis via lovastatin could block the production of VIBs induced by grass carp *vdra* or *vdrb*, we next considered whether grass carp *vdra* or *vdrb* regulated gene expression involved in cholesterol synthesis and uptake. A sterol regulatory element binding protein 1 (SREBP1) transcription factor activates the transcription of the low density lipoprotein receptor (LDLR) gene (29). However, the liver X receptors (LXRs) regulate LDLR-dependent cholesterol uptake through a pathway independent of SREBPs (30). In the absence of infection, knockdown of grass carp *vdra* or *vdrb* inhibited the transcriptions of *lxra* and *ldlr*. The mRNA levels of *srebf1*, *fdft1*, and *hmgcr*, which are involved in cholesterol biosynthesis, were also downregulated by knockdown of grass carp *vdra* or *vdrb*, especially by double knockdown of grass carp *vdra* and *vdrb*. GCRV infection significantly induced the expressions of *lxra*, *ldlr*, *srebf1*, *fdft1*, and *hmgcr*; however, knockdown of grass carp *vdra* or *vdrb* attenuated GCRV-induced expressions of these genes involved in cholesterol synthesis and uptake. The GCRV-induced expressions of *ldlr*, *srebf1*, *fdft1*, and *hmgcr* were even completely blocked by the double knockdown of grass carp *vdra* and *vdrb* (Fig. 7E).

All these data suggest that grass carp *vdra* and *vdrb* in the absence of VitD promote GCRV replication via a manner independent of the RLR antiviral signaling pathway but dependent of cholesterol biosynthesis.

### VitD activates the RLR antiviral signaling pathway via the Vdra-Rxrbb heterodimer.

**FIG 8:**
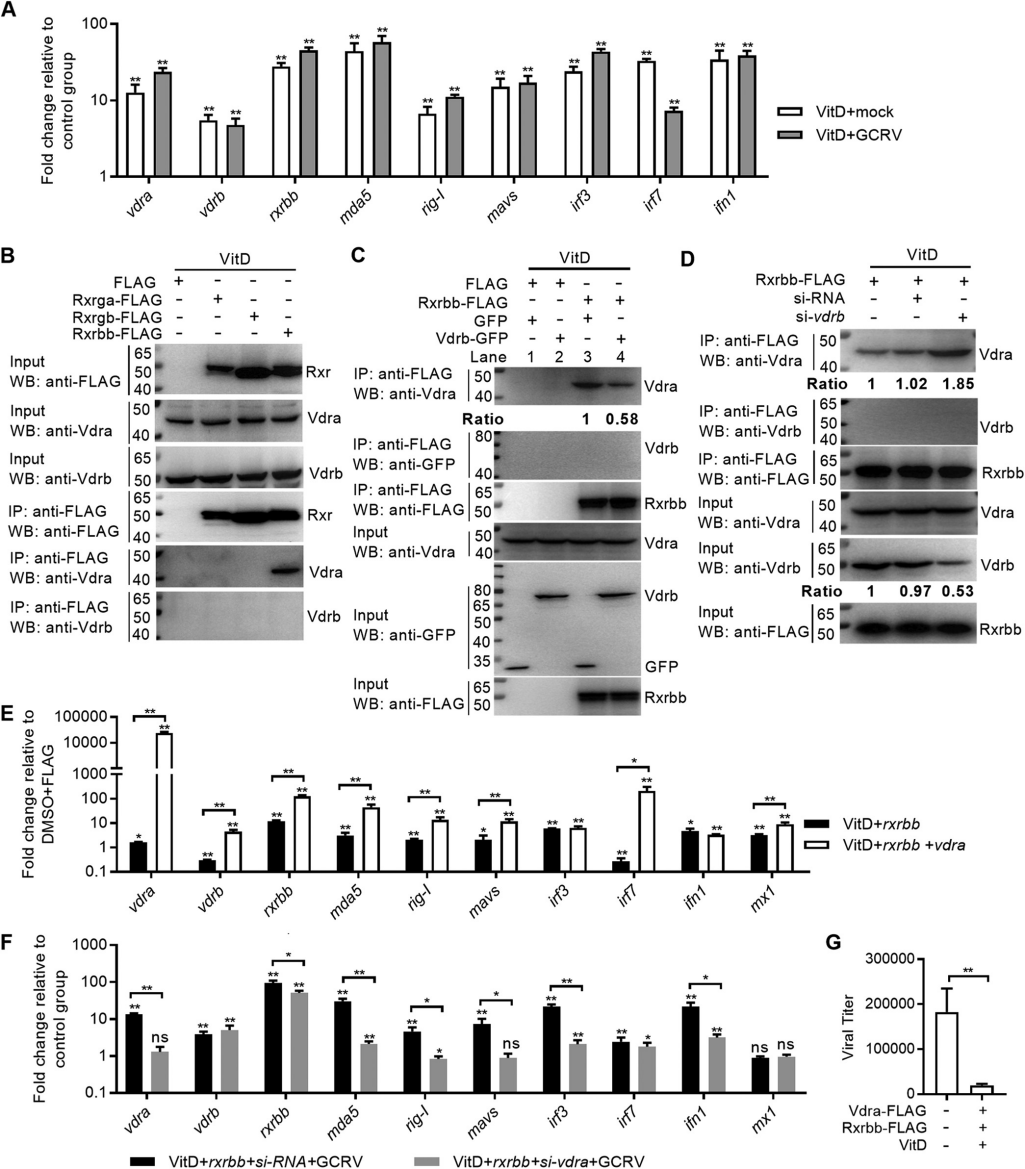
VitD activates the RLR antiviral signaling pathway via the Vdra-Rxrbb heterodimer. (A) The effects of VitD on the expressions of antiviral genes with or without GCRV infection. CIK cells grown in 6-well plates were transfected with 80 ng VitD. After 24 h posttransfection, the cells were infected with GCRV at an MOI of 1 or left untreated. At 24 hpi, these cells were collected and used for qRT-PCR. (B) The interaction between Vdr and Rxr in the presence of VitD. CIK cells seeded in 10-cm^2^ dishes were transfected with 10 μg FLAG, *rxrga*-FLAG, *rxrgb*-FLAG, or *rxrbb*-FLAG or 400 ng VitD. After 48 h posttransfection, the cells were harvested and used for co-IP. (C) The effect of grass carp Vdrb on the formation of Vdra-Rxrbb heterodimer in the presence of VitD. CIK cells seeded in 10-cm^2^ dishes were transfected with 10 μg FLAG, *rxrbb*-FLAG, GFP, or *vdrb*-GFP or 400 ng VitD with the indicated combination. After 48 h posttransfection, the cells were harvested and used for co-IP. (D) The effect of grass carp *vdrb* knockdown on the formation of Vdra-Rxrbb heterodimer in the presence of VitD. CIK cells seeded in 10-cm^2^ dishes were transfected with 10 μg *rxrbb*-FLAG, 100 nM siRNA, si-*vdrb*, or 400 ng VitD with the indicated combination. After 48 h posttransfection, the cells were harvested and used for co-IP. For C and D, protein bands were quantified by Image J. (E) The effect of grass carp *vdra* on the transcriptional regulation of antiviral genes involved in RLR signaling pathway in the presence of VitD and *rxrbb*. CIK cells seeded overnight in 6-well plates were transfected with 1,000 ng FLAG, *rxrbb*-FLAG, or *vdra*-FLAG or 80 ng VitD with the indicated combination. After 48 h posttransfection, the cells were collected and used for qRT-PCR. (F) The effect of grass carp *vdra* knockdown on the transcriptional regulation of antiviral genes involved in RLR signaling pathway in the presence of VitD and *rxrbb* with GCRV infection. CIK cells seeded overnight in 6-well plates were transfected with 1,000 ng FLAG, *rxrbb*-FLAG, 100 nM siRNA, si-*vdra*, DMSO, or 80 ng VitD with the indicated combination. After 24 h posttransfection, these cells were infected with GCRV at an MOI of 1 or left untreated. At 24 hpi, these cells were collected and used for qRT-PCR. (G) The effect of VitD-activated Vdra-Rxrbb heterodimer on the GCRV replication. CIK cells grown in 24-well plates were cotransfected with 20 ng VitD, 800 ng *vdra*-FLAG, and *rxrbb*-FLAG, with CIK cells transfected with DMSO and FLAG as the control group. After 24 h posttransfection, the cells were infected with GCRV at an MOI of 1 for 24 h and used for determining GCRV titers. For A, the asterisks above the error bars indicated statistical significance using the group in the absence of both VitD transfection and GCRV infection as the control group. For E, the asterisks above the error bars indicated statistical significance using the group transfected with DMSO and FLAG as the control group. For F, the asterisks above the error bars indicated statistical significance using the group transfected with DMSO, FLAG, and siRNA in the absence of GCRV infection as the control group. For E to G, the asterisks above the bracket indicated statistical significance between the two groups connected by the bracket. *, *P < *0.05; **, *P < *0.01; ns, not significant.

The mechanisms of how VitD interfered with GCRV replication were further investigated. We first investigated the role of VitD in transcriptional activation of these genes involved in the RLR antiviral signaling pathway. As shown in Fig. 8A, VitD enhanced the transcriptions of all tested genes, including *vdra*, *vdrb*, *rxrbb*, *mda5*, *rig-I*, *mavs*, *irf3*, *irf7*, and *ifn1*.

In mammals, the VDR-RXR heterodimer interacts with different types of VDREs present in the promoter region of target genes, and VitD can regulate the transcription of many genes via the VDR-RXR heterodimer (31). We next determined whether grass carp Vdra or Vdrb interacted with Rxr in the presence of VitD. CIK cells were cotransfected with VitD and tagged with Rxrbb, Rxrga, Rxrgb, or FLAG. The results from the coimmunoprecipitation (co-IP) assay showed that only Vdra interacted with Rxrbb in the presence of VitD (Fig. 8B). The possible effect of Vdrb on the formation of the Vdra-Rxrbb heterodimer was further investigated. CIK cells were cotransfected with VitD and indicated plasmids. When Rxrbb-FLAG was overexpressed, the anti-FLAG-M2 affinity gel-immunoprecipitated Rxrbb failed to associate with grass carp Vdrb (lane 4 using anti-GFP antibody for immunoprecipitation [IP] product in Fig. 8C), but Rxrbb could associate with the endogenous Vdra (Lane 3 using anti-Vdra antibody for IP product in Fig. 8C). The association between the endogenous Vdra and Rxrbb was impaired by grass carp Vdrb (lanes 3 and 4 using anti-Vdra antibody for the IP product in Fig. 8C). Finally, we determined whether the knockdown of grass carp *vdrb* promoted the ability of Vdra to interact with Rxrbb in the presence of VitD. The decrease of Vdrb expression did promote the interaction between Vdra and Rxrbb (Fig. 8D).

Since VitD promoted the formation of the Vdra-Rxrbb heterodimer, the effect of *vdra* in the transcriptional activation of the RLR antiviral signaling pathway via the VitD-Vdra-Rxrbb complex was determined. Compared with the group transfected with VitD and *rxrbb*, overexpression of grass carp *vdra* further heightened the transcription of *vdrb*, *rxrbb*, *mda5*, *rig-I*, *mavs*, *irf7*, and *mx1*, with the fold change increase of 4.46-fold, 126.67-fold, 44.67-fold, 13.40-fold, 11.59-fold, 208.63-fold, and 8.93-fold, respectively (Fig. 8E). The effect of grass carp *vdra* on the transcriptional regulation of genes involved in the RLR signaling pathway in the presence of VitD and *rxrbb* was further investigated under the condition of GCRV infection. Compared with the group transfected with VitD, *rxrbb*, and siRNA, knockdown of grass carp *vdra* significantly impaired the transcriptional levels of *rxrbb*, *mda5*, *rig-I*, *mavs*, *irf3*, and *ifn1* during GCRV infection (Fig. 8F). Lastly, the effect of the Vdra-Rxrbb heterodimer induced by VitD on GCRV replication was determined. Compared with the control group transfected with FLAG and dimethyl sulfoxide (DMSO), the viral titer was significantly decreased in the CIK cells cotransfected with VitD, Vdra, and Rxrbb (Fig. 8G).

Taken together, these results suggest that grass carp Vdra but not Vdrb in the presence of VitD can form the heterodimer with Rxrbb and induces the transcription of those genes involved in the RLR antiviral signaling pathway for inhibiting GCRV infection.

### VitD-activated Vdra-Vdrb heterodimer attenuates the transcriptions of the RLR antiviral signaling pathway induced by VitD.

**FIG 9:**
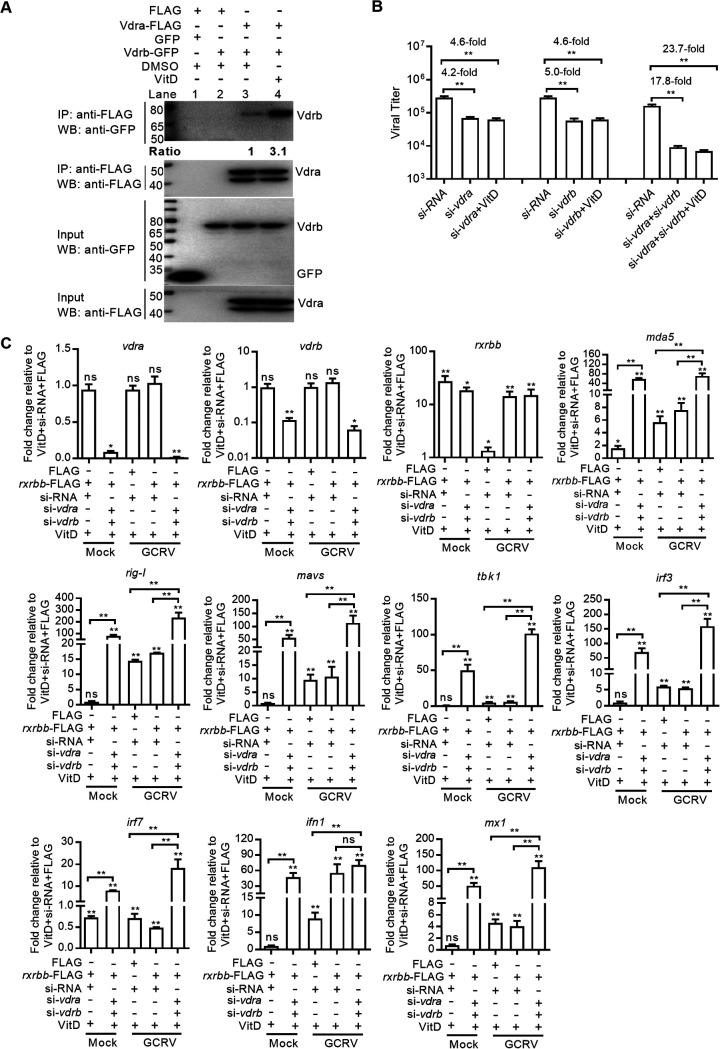
VitD-activated Vdra-Vdrb heterodimer attenuates the transcriptions of the RLR antiviral signaling pathway induced by VitD. (A) The interaction between grass carp Vdra and Vdrb with or without the VitD. CIK cells seeded in 10-cm^2^ dishes were transfected with 10 μg FLAG, *vdra*-FLAG, GFP, *vdrb*-GFP, DMSO, or 400 ng VitD with the indicated combination. After 48 h posttransfection, the cells were harvested and used for co-IP. (B) Determination of GCRV titers for knockdown of grass carp Vdra or/and Vdrb in CIK cells with or without the VitD. CIK cells grown in 24-well plates were transfected with 100 nM siRNA, si-*vdra* or/and si-*vdrb*, or 20 ng VitD with the indicated combination. After 24 h posttransfection, the cells were infected with GCRV at an MOI of 1 for 24 h and used for determining GCRV titers. (C) The effects of double knockdown of grass carp *vdra* and *vdrb* on the transcriptions of antiviral genes involved in the RLR signaling pathway with or without the GCRV infection. CIK cells grown in 6-well plates were transfected with 1,000 ng FLAG, *rxrbb*-FLAG, 100 nM siRNA, si-*vdra* or/and si-*vdrb*, and 80 ng VitD with the indicated combination. After 24 h posttransfection, the cells were infected with GCRV at an MOI of 1 or left untreated. At 24 hpi, these cells were collected and used for qRT-PCR. For C, the asterisks above the error bars indicated statistical significance using the group transfected with VitD, siRNA, and FLAG plasmid in the absence of GCRV infection as the control group. For B and C, the asterisks above the bracket indicated statistical significance between the two groups connected by the bracket. *, *P < *0.05; **, *P < *0.01; ns, not significant.

Since VitD affects the formation of the Vdra-Rxrbb heterodimer, we were interested to know the possible effect of VitD on the formation of Vdra-Vdrb complex. In the absence of VitD, grass carp Vdrb formed a weak heterodimer with Vdra (lane 3 using anti-GFP antibody for IP product in Fig. 9A). In the presence of VitD, the formation of the Vdra-Vdrb complex was obviously increased (lanes 3 and 4 using anti-GFP antibody for IP product in Fig. 9A). The effects of the VitD and Vdra-Vdrb complex on GCRV replication was further investigated. Compared with a knockdown of grass carp *vdra* or *vdrb*, the double knockdown of grass carp *vdra* and *vdrb* in the presence of VitD showed the strongest protective effect against GCRV infection with the lowest viral load (Fig. 9B).

The above results showed that in the absence of VitD, grass carp *vdra* and *vdrb* during GCRV infection had no significant correlation with the RLR antiviral signaling pathway (Fig. 7D). Since VitD promoted the formation of the Vdra-Vdrb complex, the effects of the Vdra-Vdrb complex on the transcriptional regulation of the RLR antiviral signaling pathway mediated by VitD were further investigated. Different from the above results in the absence of VitD, double knockdown of grass carp *vdra* and *vdrb* enormously induced the expressions of *mda5*, *rig-I*, *mavs*, *tbk1*, *irf3*, *irf7*, *ifn1*, and *mx1* in the presence of VitD. Compared with the group cotransfected with VitD, siRNA, and FLAG, overexpression of *rxrbb* had no obvious effects on the transcriptions of *rig-I*, *mavs*, *tbk1*, *irf3*, *ifn1*, and *mx1* and slightly regulated the transcriptions of *mda5* (1.6-fold increase) and *irf7* (1.4-fold decrease). However, compared with the group cotransfected with VitD, siRNA, and FLAG, the upregulated fold changes by the knockdown of grass carp *vdra* and *vdrb* in the presence of VitD during GCRV infection were 71.5-fold, 235.2-fold, 113.7-fold, 101.5-fold, 159.5-fold, 18.4-fold, 70.4-fold, and 110.3-fold for *mda5*, *rig-I*, *mavs*, *tbk1*, *irf3*, *irf7*, *ifn1*, and *mx1*, respectively. In the presence of VitD and *rxrbb* during GCRV infection, the double knockout of grass carp *vdra* and *vdrb* significantly induced the transcriptions of *mda5*, *rig-I*, *mavs*, *tbk1*, *irf3*, *irf7*, and *mx1* (Fig. 9C).

Taken together, these results suggest that VitD also activates a Vdra-Vdrb heterodimer as a corepressor, which attenuates the transcriptions of the RLR antiviral signaling pathway induced by VitD for balancing GCRV infection mediated by the VitD-activated Vdra-Rxrbb heterodimer as a coactivator.

### VitD abolishes the negative regulation of grass carp Vdrs during GCRV infection in a dose-dependent manner.

**FIG 10:**
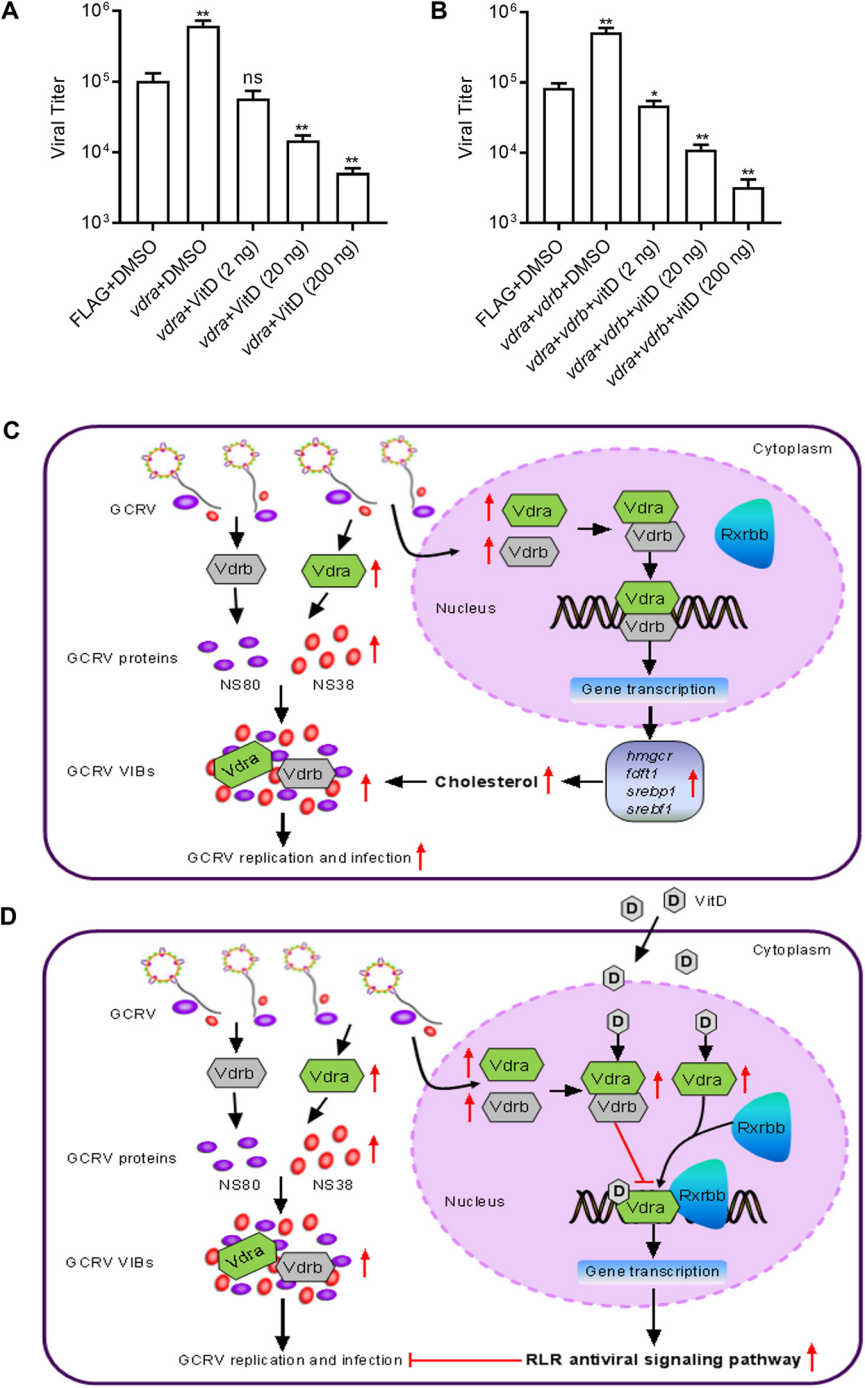
The effects of grass carp *vdrs* and VitD during GCRV infection. (A) VitD abolishes the negative regulation of grass carp *vdra* during GCRV infection. CIK cells grown in 24-well plates were transfected with 800 ng FLAG or *vdra*-FLAG with or without 2 to ~200 ng VitD. After 24 h posttransfection, the cells were infected with GCRV at an MOI of 1 for 24 h and used for determining GCRV titers. (B) VitD abolishes the negative regulation of grass carp *vdra* and *vdrb* during GCRV infection. CIK cells grown in 24-well plates were transfected with 800 ng FLAG, *vdra*-FLAG, and *vdrb*-FLAG with or without 2 to ~200 ng VitD. After 24 h posttransfection, the cells were infected with GCRV at an MOI of 1 for 24 h and used for determining GCRV titers. (C) Proposed model illustrating the pivotal roles of grass carp *vdrs* in the negative regulation of GCRV infection via *hmgcr*-dependent cholesterol synthesis. (D) Proposed model illustrating the pivotal roles of VitD in attenuating or preventing the negative regulation of grass carp *vdrs* on GCRV infection via balancing the transcriptional regulation of the RLR antiviral signaling pathway mediated by the Vdra-Rxrbb and Vdra-Vdrb heterodimers. For A and B, the asterisks above the error bars indicated statistical significance using the group transfected with DMSO and FLAG plasmid as the control group. *, *P < *0.05; **, *P < *0.01; ns, not significant.

Both VitD and Vdra are involved in the formation of Vdra-Rxrbb and Vdra-Vdrb heterodimers. VitD inhibited GCRV replication, but grass carp Vdra or Vdrs promoted GCRV replication. We were interested to determine the effects of grass carp Vdra or Vdrs on GCRV replication in the presence of different concentrations of VitD. As shown in Fig. 10A and B, the negative regulation of grass carp Vdra or Vdrs during GCRV infection is completely abolished by transfection with 2 to ~200 ng VitD. Compared with the control group transfected with FLAG and DMSO, the viral titers were significantly increased in the group transfected with *vdra* and DMSO, had no obvious change in the group transfected with *vdra* and 2 ng VitD, and decreased in the group transfected with *vdra* and 20 ng or 200 ng VitD (Fig. 10A). Compared with the control group transfected with FLAG and DMSO, the viral titers were significantly decreased in all the groups transfected with *vdra* and *vdrb* and 2 ng to 200 ng VitD (Fig. 10B).

## DISCUSSION

VitD and its receptor VDR have increasingly been recognized for their effects on the regulation of the innate and adaptive immune pathways as well as several bacterial and viral infections (32). Previous study showed that mammalian VDR interacted directly with the VDRE cluster in the core promoter of hepatitis B virus (HBV) to suppress virus activity (33). In the current study, we revealed the functional properties of piscine *vdra* and *vdrb* in response to GCRV infection. Our work identified grass carp *vdrs* as the potent host factors utilized by GCRV via interacting with GCRV proteins and promoting their expressions. Our results further demonstrated that grass carp *vdrs* in the absence of VitD were beneficial for the production of VIBs and the negative regulation of *vdrs* in GCRV infection could be abolished by VitD. In addition, the present study also established a novel connection between *vdr*- and *hmgcr*-dependent cholesterol synthesis in promoting the production of VIBs.

VitD deficiency is associated with an increased occurrence of several viral diseases, such as hepatitis, influenza viruses, respiratory syncytial virus (RSV), human immunodeficiency virus (HIV), COVID-19, and AIDS (32, 34). Several studies have indicated that double-stranded DNA (dsDNA) viruses, such as HBV and single-stranded RNA (ssRNA) virus (such as hepatitis C virus [HCV]), can inhibit the VitD signaling pathway to attenuate the immune response (34–37). Although VDR mRNA and protein levels are significantly repressed by HBV, Epstein-Barr virus (EBV), cytomegalovirus (CMV). and HIV, downregulation of VDR expression is not universally associated with viral infections (34, 38–40). HIV triggers HIV-associated nephropathy through downregulation of VDR (41). In HepAD38 cells, high levels of HBV expression were found to be associated with a decrease in the levels of VDR transcripts (34). However, CMV infection, which downregulated VDR expression, was not inhibited by the biologically active metabolites of VitD (40). In sharp contrast with the above studies, the present study demonstrated a correlation between high *vdr* expression and high viral replication of GCRV (Fig. 3B and C). The rapid, pronounced, and sustained upregulations of grass carp *vdrs* during GCRV infection were observed (Fig. 2C and D). We also validated the increased protein levels of grass carp Vdrs during GCRV infection (Fig. 2E). Especially, low levels of *vdra* or/and *vdrb* significantly inhibited GCRV replication and infection, and vice versa (Fig. 3B). In the case that both grass carp *vdra* and *vdrb* were expressed at low levels, the viral load of GCRV was lower (Fig. 9B). All these data suggest functional differences of VDR in the different species or in response to different viruses.

Many viruses need to modify host cholesterol homeostasis to fulfill the demands of their life cycles. In vertebrate cells, cholesterol homeostasis is tightly regulated in a feedback mechanism via these transcription factors termed sterol-regulatory element-binding proteins (SREBPs), whose transcriptionally active form is transported to the nucleus for activating the transcription of LDLR and target genes required for cholesterol biosynthesis, including HMGCR (42–44). West Nile virus (WNV) was found to increase cholesterol synthesis for promoting its replication, whereas reducing cholesterol production by HMGCR inhibition drastically hampered virus replication (43). Inhibition of cholesterol synthesis by statins (atorvastatin, lovastatin, and simvastatin) or HMGCR knockdown had a significant inhibitory effect on rotavirus replication (45). However, the Kaposi’s sarcoma-associated herpesvirus (KSHV), whose microRNAs (miRNAs) target enzymes in the mevalonate pathway to decrease cholesterol synthesis, takes the opposite strategy to promote its replication (46). Although a previous study has shown that treatment with methyl-beta-cyclodextrin (MβCD), which is known to extract cholesterol from cell membrane, can inhibit the internalization of GCRV (47), the importance of cholesterol metabolism for GCRV replication remains largely obscure. In this study, we found that GCRV infection induced the expression of many genes involved in cholesterol biosynthesis (Fig. 7E). Of note, *srebf1*, *fdft1*, *hmgcr*, and *ldlr* were significantly upregulated in CIK cells infected with GCRV (Fig. 7E). Furthermore, inhibition of cholesterol synthesis by lovastatin, an inhibitor of *hmgcr*, impaired the production of GCRV VIBs (Fig. 6G to J). These data reveal that GCRV replication has a close relationship with *hmgcr*-dependent cholesterol synthesis. The present study also confirms that *vdr* is vital for *hmgcr*-dependent cholesterol synthesis and GCRV replication. Knockdown of grass carp *vdra* or *vdrb* enormously impaired the transcription of *srebf1*, *fdft1*, *hmgcr*, and *ldlr*, especially by double knockdown of grass carp *vdra* and *vdrb* (Fig. 7E). The GCRV-induced expression of those genes involved in cholesterol biosynthesis was completely blocked and even impaired by double knockdown of grass carp *vdra* and *vdrb*, along with the lowest viral loads and the strongest protection against GCRV infection (Fig. 7E and 9B). These findings suggest that GCRV utilizes host *vdrs* to increase *hmgcr*-dependent cholesterol synthesis for promoting its replication.

The active translation of many viruses occurs within viral factories or VIBs, which are important functional sites for viral replication (48). For HCV, the membranous web (MW) contains the sites of viral replication and assembly, which limit the access of pattern recognition receptors to compartments within the MW via components of the nuclear transport machinery, thereby preventing the activation of cellular innate immune responses (49). However, for human rhinoviruses (HRVs), lipid droplet-derived cholesterol and lipid kinase PI4K3b are enriched on HRV replication sites. HRV uses the host PI4K3b-OSBP1-SacI-PITPb cycle along with storage cholesterol to drive its replication (50). For GCRV, previous studies have revealed that the viral NS38 and NS80 are two main proteins to form the cytoplasmic VIBs (51–53). NS80 is the crucial protein required for the formation of VIBs, and NS38 is retained within VIBs by interacting with NS80 (53). Furthermore, NS38 is required for GCRV replication via interaction with viral core structural proteins and host cellular component eIF3A (54). The NS38 and NS80 of GCRV are also found to hijack host TBK1 and IRF3 into the cytoplasmic VIBs for decreasing the formations of TBK1-containing functional complexes and inhibiting the translocation of IRF3 into the nucleus (55). Among bile acid receptors, which regulate numerous metabolic pathways in the host, VDR is the only receptor with mVDR and nVDR. GCRV infection induced the accumulation of grass carp Vdrs both in the cytoplasm and nucleus (Fig. 2I). Unexpectedly, grass carp Vdrs were utilized by GCRV for promoting the expressions of GCRV proteins and the production of VIBs (Fig. 5, Fig. 6A to F). Inhibition of cholesterol synthesis by lovastatin completely blocked the effects of grass carp Vdrs in promoting the production of VIBs (Fig. 6G to J). Therefore, GCRV uses the *vdrs* and cholesterol to benefit its replication in the absence of VitD.

VitD exerts its pleiotropic biological effects mainly via activation of the VDR. Similar to the observed discrepancy of the association between VDR expression and clinical outcomes of different viral infections, the inconsistent results of VitD or its metabolite supplementation in response to viral infection were also observed. It was reported previously that the chemotherapeutic VitD analog EB-1089 had no significant effect on CMV replication (40). In response to HCV infection, VitD as well as its structurally closely related analogs EB1089 and paricalcitol had no significant effect on viral replication; however, 25(OH)D_3_ inhibited the virus assembly but not entry or replication. The structurally related VitD analogs oxacalcitriol, calcipotriol, and tacalcitol suppressed HCV replication in a VDR-dependent manner (56, 57). Daily treatment with 25(OH)D_3_ or VitD did not affect viral clearance in response to H9N2 viral infection (9, 58). However, oral supplementation of 25(OH)D3 significantly reduced virus replication and clinical manifestations of influenza A (H1N1) infection in mice (59). In our study, we observed the direct antiviral effect of VitD alone and substantially increased expressions of antiviral genes involved in the RLR antiviral signaling pathway *in vitro* (Fig. 3D and E, Fig. 8A). This finding is in line with a previous study by Gal-Tanamy et al. (60), which reported that the antiviral effect of VitD alone was associated with the induction of the interferon signaling pathway.

In conclusion, our findings reveal that piscine *vdr* signaling plays a quite different role during GCRV infection in the absence or presence of VitD (Fig. 10C and D). In the absence of VitD, the nuclear Vdra and Vdrb fail to heterodimerize with Rxrbb but form the Vdra-Vdrb dimer to induce the transcription of many genes involved in cholesterol biosynthesis (Fig. 7A to C, Fig. 7E). Fueled by cholesterol, the cytoplasmic Vdra and Vdrb are utilized by GCRV nonstructural proteins NS38 and NS80 via protein-protein interactions and promote the production of VIBs, which are beneficial for the replication and infection of GCRV (Fig. 4 and 6). The mechanism by which grass carp *vdra* and *vdrb* in the absence of VitD negatively regulate GCRV infection depends on cholesterol biosynthesis but is independent of the RLR antiviral signaling pathway (Fig. 7D and E, Fig. 10C). In the presence of VitD, grass carp Vdra can form the heterodimer with Rxrbb (Fig. 8B to D). VitD activates the RLR antiviral signaling pathway via the Vdra-Rxrbb heterodimer for inhibiting GCRV infection (Fig. 8E to G). Furthermore, the VitD-activated Vdra-Vdrb heterodimer attenuates the transcriptions of the RLR antiviral signaling pathway induced by VitD (Fig. 9). A balance between coactivators, such as Vdra-Rxrbb, or corepressors, such as Vdra-Vdrb, in affecting the transcriptional regulation of the RLR antiviral signaling pathway may determine the outcome of the infection (Fig. 10D). The results provided in this study lay a foundation for elucidating the role of piscine bile acid receptor *vdr* in the infectious disease and offer a novel mechanistic insight into the therapeutic efficacy of supplemental VitD for protection against GCRV infection.

## MATERIALS AND METHODS

### Cells, virus, and plasmids.

CIK cells were grown in minimum essential medium (MEM) supplemented with 10% fetal bovine serum (FBS). GCRV-873 was propagated in CIK cells using MEM supplemented with 2% FBS. Plasmids used in this study, including the pTurboGFP vector (Evrogen) and p3×FLAG-CMV-14 expression vector (Sigma-Aldrich Co. LLC), were stored in our laboratory. *vdra*-FLAG, *vdrb*-FLAG, *rxrga*-FLAG, *rxrgb*-FLAG, and *rxrbb*-FLAG were obtained using the primer pairs *vdra*-F1/*vdra*-R1, *vdrb*-F1/*vdrb*-R1, *rxrga*-F1/*rxrga*-R1, *rxrgb*-F1/*rxrgb*-R1, and *rxrbb*-F1/*rxrbb*-R1, respectively, and were cloned into the p3×FLAG-CMV-14 vector. *vdrb*-GFP was obtained using the primer pairs *vdrb*-F2/*vdrb*-R2 and was cloned into the pTurboGFP-N vector. The primers used for plasmid constructs are listed in Table S1 in the supplemental material.

### Antibodies and reagents.

The anti-FLAG mouse monoclonal antibody (number F3165), anti-pTurboGFP rabbit polyclonal antibody (number AB513), and anti-GAPDH mouse monoclonal antibody (number 60004-1-Ig) were purchased from Sigma-Aldrich, Everogen, and Proteintech, respectively. The anti-NS38 polyclonal mouse antibody and anti-NS80 polyclonal rabbit antibody against the GCRV-873 strain were prepared previously (7–10). Lovastatin (S2061), VitD (S1466), MG132 (S2619), and 3-MA (S2767) were purchased from Selleck (Shanghai, China). Goat-anti-mouse immunoglobulin-horseradish peroxidase (Ig-HRP) conjugate secondary antibody, goat-anti-rabbit Ig-HRP conjugate secondary antibody, Alexa Fluor 488-conjugated secondary Ab against mouse IgG, Alexa Fluor 594-conjugated secondary Ab against rabbit IgG, 4′,6-diamidino-2-phenylindole (DAPI), Subcellular Protein Fractionation kit, Lipofectamine 2000, and protease inhibitor cocktail were purchased from Thermo Fisher Scientific. The FLAG immunoprecipitation kit and chloroquine (C6628) were purchased from Sigma-Aldrich.

### Knockdown of grass carp *vdra* or *vdrb* by siRNA.

Transient knockdown of grass carp *vdra* or *vdrb* was achieved by transfection of siRNA targeting the mRNAs of grass carp *vdra* or *vdrb*. Three siRNA sequences, including si-*vdra*1 (5′-GACACTAAGCTGAACTTCA-3′), si-*vdra*2 (5′-GCATGATGAAGGAATTCAT-3′), si-*vdra*3 (5′-GAGGAACATGTGCTGCTGA-3′), si-*vdrb*1 (5′-CACCATCACTAAAGATAAC-3′), si-*vdrb*2 (5′-GCAACATCATTGACACGCT-3′), and si-*vdrb*3 (5′-GTGCTATAGAAGTGATCAT-3′), targeting different regions of grass carp *vdra* or *vdrb*, were synthesized by Ribobio (Guangzhou, China). To verify the silencing efficiencies of these siRNAs, CIK cells grown in 6-well plates were transfected with 100 nM siRNA (control), si-*vdra*1, si-*vdra*2, si-*vdra*3, si-*vdrb*1, si-*vdrb*2, or si-*vdrb*3. After 24 h posttransfection, these cells were harvested and used for qRT-PCR.

### Sequence analysis.

A multiple sequence alignment was generated using ClustalO and modified with the BoxShade software (https://junli.netlify.app/apps/boxshade/). The protein domains of Vdr were predicted using the Simple Modular Architecture Research Tool (SMART) (http://smart.embl-heidelberg.de/). The phylogenetic tree was constructed with MEGA 7.0 software (Jones-Taylor-Thornton [JTT] model), with the bootstrap setting as 10,000 to test the reliability of branching.

### GCRV infection in grass carp.

Grass carp (30 ± 2 g) were purchased from a local fish farm (Wuhan, China). All fish were acclimatized at 25 ± 2°C for 14 days in aerated tanks and fed with a commercial diet twice daily until 24 h before the challenge experiment. All experiments complied with the national regulations and local guidelines for the use of animals for research. For tissue sampling, fish were anaesthetized with MS-222 and killed by cutting the spinal cord. Seven tissues, including liver, brain, skin, intestine, gill, kidney, and spleen, from three healthy fish were collected under anesthesia with MS222 (0.1 g/L). The collected tissues were immediately frozen in liquid nitrogen and then stored at −80°C for RNA extraction and qRT-PCR. For GCRV infection in grass carp, 30 healthy fish were divided randomly into two groups. One group was injected intraperitoneally (i.p.) with GCRV (300 μL, 1 × 10^8^ PFU/mL) and the other group was injected with phosphate-buffered saline (PBS) (control). Four tissues, including intestine, gill, kidney, and spleen, from 3 fish of each group were collected at 6, 24, 48, and 72 hpi and used for RNA extraction and qRT-PCR. The spleens from the uninfected grass carp and the infected grass carp at 6 and 24 hpi were used for protein extraction and Western blotting.

### Immunofluorescence assays.

To determine the effect of GCRV infection on the expression and subcellular localizations of grass carp Vdra and Vdrb, CIK cells plated onto coverslips in 24-well plates were infected with GCRV at a multiplicity of infection (MOI) of 1 or left untreated. At 6 and 18 hpi, the cells were washed twice with PBS and fixed with 4% paraformaldehyde (PFA) for 1 h. After permeabilization and blocking, the cells were washed three times with PBS, incubated with anti-Vdra (1:2,000) or anti-Vdrb (1:2,000) antibodies for 2 h at 37°C, and then incubated with an Alexa Fluor 594-conjugated secondary Ab against rabbit IgG (1:500).

To determine the possible colocalization between grass carp Vdra or Vdrb and the exogenous NS38 or NS80 nonstructural protein of GCRV, CIK cells plated onto coverslips in 24-well plates were transfected with 800 ng NS38-FLAG or NS80-FLAG. After 24 h posttransfection, the cells were washed twice with PBS and fixed with 4% paraformaldehyde (PFA) for 1 h. After permeabilization and blocking, the cells were washed and incubated with anti-FLAG (1:5,000), anti-Vdra (1:2,000), or anti-Vdrb (1:2,000) antibodies for 2 h at 37°C and then incubated with Alexa Fluor 488-conjugated secondary Ab against mouse IgG (1:500) and Alexa Fluor 594-conjugated secondary antibody against rabbit IgG (1:500). To investigate the possible colocalization between grass carp Vdra or Vdrb and the endogenous protein of GCRV, CIK cells plated onto coverslips in 24-well plates were infected with GCRV at an MOI of 1 or left untreated. At 6 and 24 hpi, the cells were washed twice with PBS and fixed with 4% PFA for 1 h. After permeabilization and blocking, the cells were washed and incubated with anti-NS38 (1:2,000) or anti-VP3 (1:1,000) and anti-Vdra (1:2000) or anti-Vdrb (1:2000) antibodies for 2 h at 37°C and then incubated with Alexa Fluor 488-conjugated secondary Ab against mouse IgG (1:500) and Alexa Fluor 594-conjugated secondary antibody against rabbit IgG (1:500).

To determine the effects of grass carp Vdrs on the production of VIBs, CIK cells plated onto coverslips in 24-well plates were transfected with 800 ng FLAG, *vdra-*FLAG, *vdrb*-FLAG, 100 nM siRNA, si-*vdra*, or si-*vdrb*, respectively. After 24 h, the cells were infected with GCRV at an MOI of 1. At 24 hpi, the cells were collected, washed twice with PBS, and fixed with 4% PFA for 1 h. After permeabilization and blocking, the cells were washed three times with PBS; incubated with anti-FLAG (1:5,000), anti-Vdra (1:2,000), or anti-Vdrb (1:2,000) and anti-NS38 (1:2000) for 2 h at 37°C; and then incubated with Alexa Fluor 488-conjugated secondary Ab against mouse IgG (1:500) and Alexa Fluor 594-conjugated secondary Ab against rabbit IgG (1:500).

To determine the effects of lovastatin on the production of VIBs induced by grass carp Vdrs, CIK cells plated onto coverslips in 24-well plates were transfected with 800 ng FLAG, *vdra*-FLAG, or *vdrb*-FLAG. After 24 h, the cells were infected with GCRV at an MOI of 1. Following adsorption, the cells were washed with PBS to remove nonadsorbed virions. Then, the infected cells were incubated with 80 μM lovastatin or left untreated. After 24 h of treatment, the cells were washed twice with PBS and fixed with 4% PFA for 1 h. After permeabilization and blocking, the cells were washed three times with PBS, incubated with anti-FLAG (1:5,000) and anti-NS38 (1:2,000) for 2 h at 37°C, and then incubated with Alexa Fluor 488-conjugated secondary Ab against mouse IgG (1:500) and Alexa Fluor 594-conjugated secondary Ab against rabbit IgG (1:500).

For all the immunofluorescence assays, DAPI staining was applied to detect the cell nucleus. After each incubation step, cells were washed with PBS. Finally, the coverslips were washed and the images were obtained using a SP8 Leica laser confocal microscopy imaging system.

### Preparation of nuclear and cytoplasmic fractions.

To investigate the effect of GCRV infection on the expression and subcellular localizations of grass carp Vdra and Vdrb by Western blotting, 1 × 10^6^ CIK cells cultured in 6-well plates overnight were infected with GCRV at an MOI of 1 or left untreated. At 24 hpi, the cells were harvested and used for preparation of nuclear and cytoplasmic extract using a subcellular protein fractionation kit. The purity of the fractions was determined by Western blotting using polyclonal anti-tubulin (1:5,000; ab6046; Abcam) and anti-HDAC1 (1:5,000; ab41407; Abcam) antibodies. The protein expressions of grass carp Vdra and Vdrb were examined using polyclonal anti-Vdra (1:2000) and anti-Vdrb (1:2,000) antibodies. The nuclear and cytoplasmic proteins were subjected to 10% SDS-PAGE and transferred to polyvinylidene difluoride (PVDF) membranes. After each wash step with Tris-buffered saline with Tween 20 (TBST), the membranes were incubated with primary antibodies overnight at 4°C and then incubated with goat anti-mouse Ig-HRP conjugate secondary Ab (1:5,000) or goat-anti-rabbit Ig-HRP conjugate secondary Ab (1:5,000) for 2 h at room temperature.

### GCRV infection in CIK cells.

To determine the role of the overexpression of grass carp *vdra* or *vdrb* in GCRV infection, CIK cells grown in 24-well plates were transfected with 800 ng FLAG empty plasmid, *vdra*-FLAG, or *vdrb*-FLAG. To verify the effect of knock down of grass carp *vdra* or *vdrb* in GCRV infection, CIK cells grown in 24-well plates were transfected with 100 nM siRNA, si-*vdra*, or si-*vdrb*. After 24 h posttransfection, the cells were infected with GCRV at an MOI of 1 at 25°C or left untreated. To investigate the effects of the transfection of VitD or VitD addition to the medium on the GCRV replication mediated by grass carp *vdra* or *vdrb*, CIK cells grown in 24-well plates were transfected with 800 ng FLAG, *vdra*-FLAG, or *vdrb*-FLAG, altogether with the transfection of 20 ng VitD or the addition of 200 ng VitD to the medium or were left untreated. To determine the role of VitD with the different concentrations on GCRV replication, CIK cells grown in 24-well plates were transfected with or without 2 to ~200 ng VitD. To determine the role of the VitD-activated Vdra-Rxrbb heterodimer on the GCRV replication, CIK cells grown in 24-well plates were cotransfected with 20 ng VitD, 800 ng *vdra*-FLAG, and *rxrbb*-FLAG, with CIK cells transfected with DMSO and FLAG as the control group. After 24 h posttransfection, the cells were infected with GCRV at an MOI of 1 at 25°C. To determine the effects of transfection of VitD for different times during GCRV replication, CIK cells grown in 24-well plates were transfected with 20 ng VitD. After 12, 24, and 48 h posttransfection, the cells were infected with GCRV at an MOI of 1 at 25°C. To determine the role of VitD with the different concentrations on GCRV replication mediated by grass carp *vdra* or/and *vdrb*, CIK cells grown in 24-well plates were transfected with 800 ng FLAG, *vdra*-FLAG, or/and *vdrb*-FLAG with or without 2 to ~200 ng VitD. After 24 h posttransfection, the cells were infected with GCRV at an MOI of 1 at 25°C. For the experiments mentioned above, the cells after GCRV adsorption for 1 h were washed with PBS to remove nonadsorbed virions. Then, the GCRV-infected cells were maintained in 2% FBS and MEM at 25°C for 24 h. Next, 24-well plates fixed in 4% paraformaldehyde for 1 h were stained with 1% crystal violet and photographed. The supernatants in 24-well plates were collected to measure viral titers using cytopathic effect (CPE)-based 50% tissue culture infective dose (TCID_50_). In short, viral samples were serially diluted at 1:10 in 1× PBS, and the diluted viral samples were inoculated in triplicate onto confluent monolayers of CIK cells. Infected cells were incubated at 25°C in MEM with 2% FBS for 72 h. Virus infectivity was measured by calculating the TCID_50_.

To investigate the effect of VitD on the transcription regulation of antiviral genes, CIK cells seeded overnight in 6-well plates at 1 × 10^6^ cells per well were transiently transfected with 80 ng VitD. To investigate the effect of the overexpression of grass carp *vdra* on the transcription regulation of antiviral genes via the Vdr-Rxr heterodimer in the presence of VitD, CIK cells seeded overnight in 6-well plates at 1 × 10^6^ cells per well were transiently transfected with 1,000 ng FLAG, *rxrbb*-FLAG, or *vdra*-FLAG or 80 ng VitD with the indicated combination. To investigate the effect of the knock down of grass carp *vdra* or/and *vdrb* on the transcription regulation of antiviral genes via the Vdr-Rxr heterodimer in the presence of VitD stimulation and/or GCRV infection, CIK cells seeded overnight in 6-well plates at 1 × 10^6^ cells per well were transiently transfected with 1,000 ng FLAG, *rxrbb*-FLAG, 100 nM siRNA, si-*vdra* or/and si-*vdrb*, or 80 ng VitD with the indicated combination. To investigate the effect of the knock down of grass carp *vdra* and *vdrb* on the transcription regulation of those genes involved in antiviral genes, cholesterol uptake, and biosynthesis, CIK cells seeded overnight in 6-well plates at 1 × 10^6^ cells per well were transiently transfected with 100 nM siRNA, si-*vdra*, or/and si-*vdrb*. For the above experiments, these cells after 24 h posttransfection were infected with GCRV at an MOI of 1 or were left untreated. For an expression analysis, these cells were collected at 24 hpi and used for qRT-PCR.

### qRT-PCR.

The TRIzol reagent (Thermo Fisher Scientific) was used for RNA extraction. The concentration of total RNA was determined by using a spectrophotometer (NanoDrop 2000; Thermo). RNase-free DNase I (Thermo) was used to remove genomic DNA remnants at 37°C for 30 min. The cDNA was synthesized using the RevertAid first strand cDNA synthesis kit (Thermo Fisher Scientific) according to the manufacturer’s instructions. qRT-PCR was performed on a Bio-Rad CFX96 C1000 thermal cycler using iQ SYBR green supermix (Bio-Rad, Singapore) under the following conditions: 3 min at 95°C, followed by 45 cycles of 10 s at 95°C, 15 s at 60°C, and 10 s at 72°C. All reactions were performed in triplicate and the mean value recorded. Those genes involved in the RLR antiviral signaling pathway (*mda5*, *rig-I*, *mavs*, *tbk1*, *irf3*, *irf7*, *ifn1*, and *mx1*) and the genes involved in cholesterol uptake and biosynthesis (*lxra*, *ldlr*, *srebf1*, *fdft1*, and *hmgcr*) were chosen. The housekeeping genes, *including β-actin, EF-1α*, and 18S rRNA were used for normalizing cDNA amounts. The fold changes relative to the control group were calculated using the threshold cycle (2^−ΔΔ^*^CT^*) method. All primers used for qRT-PCR are shown in Table S1 in the supplemental material.

### Coimmunoprecipitation (co-IP) assay and Western blotting.

To determine the possible interaction between grass carp Vdra or Vdrb and GCRV proteins, CIK cells seeded in 10-cm^2^ dishes were transfected with 10 μg FLAG, *vdra*-FLAG, or *vdrb*-FLAG. After 24 h posttransfection, the cells were infected with GCRV at an MOI of 1 or left untreated. At 24 hpi, the cells were collected and lysed in 600 μL IP lysis buffer containing protease inhibitor cocktail. Cellular debris was removed by centrifugation at 12,000 × *g* for 10 min at 4°C. Co-IP was performed using the FLAG-tagged protein immunoprecipitation kit according to the manufacturer’s manual. The agarose was washed six times with ice-cold washing solution, and the protein was eluted with elution buffer. Total lysate and eluted proteins were analyzed by Western blotting with anti-FLAG (1:5,000), anti-VP3 (1:1,000), anti-NS38 (1:5,000), or anti-NS80 (1:5,000) antibody.

To determine the possible interaction between grass carp Vdrs and Rxrs, CIK cells seeded in 10-cm^2^ dishes were transfected with 10 μg FLAG, *rxrga*-FLAG, *rxrgb*-FLAG, or *rxrbb*-FLAG. After 48 h posttransfection, the cells were lysed in IP lysis buffer containing a cocktail of protease inhibitors. Co-IP was performed using the FLAG-tagged protein immunoprecipitation kit. Total lysate and eluted proteins were analyzed by Western blotting with anti-FLAG, anti-Vdra (1:2,000), or anti-Vdrb (1:2,000) antibody.

To determine the effect of GCRV infection on the Vdr-rxrbb complex with or without the stimulation of VitD, CIK cells seeded in 10-cm^2^ dishes were transfected with 10 μg FLAG or *rxrbb*-FLAG. After 24 h posttransfection, the cells were infected with GCRV at an MOI of 1 or left untreated. At 24 hpi, the cells were lysed in IP lysis buffer containing a cocktail of protease inhibitors. Co-IP was performed using the FLAG-tagged protein immunoprecipitation kit. Total lysate and eluted proteins were analyzed by Western blotting with anti-FLAG, anti-VDRa, anti-VDRb, or anti-NS80 antibodies.

To investigate the possible interaction between grass carp Vdra and Vdrb with or without the stimulation of VitD, CIK cells seeded in 10-cm^2^ dishes were transfected with 10 μg FLAG, Vdra-FLAG, GFP, Vdrb-GFP, DMSO, or 400 ng VitD with the indicated combination. To determine whether grass carp Vdrb interfered with the interaction between grass carp Vdra and Rxrbb, CIK cells seeded in 10-cm^2^ dishes were transfected with 10 μg FLAG, *rxrbb*-FLAG, GFP, *vdrb*-GFP, or 400 ng VitD with the indicated combination. To determine knockdown of grass carp *vdrb* on the formation of the Vdra-Rxrbb complex in the presence of VitD, CIK cells seeded in 10-cm^2^ dishes were transfected with 10 μg *rxrbb*-FLAG, 100 nM siRNA, si-*vdrb*, or 400 ng VitD with the indicated combination. After 48 h posttransfection, the cells were lysed in IP lysis buffer containing a cocktail of protease inhibitors. Co-IP was performed using the FLAG-tagged protein immunoprecipitation kit. Total lysate and eluted proteins were analyzed by Western blotting with anti-FLAG or anti-GFP antibody.

To examine the effect of knockdown of grass carp Vdra or Vdrb on the protein degradations of GCRV proteins, CIK cells seeded in 6-well plates overnight were transfected with 100 nM siRNA, si-Vdra, or si-Vdrb. After 24 h of transfection, the cells were treated with 3-MA, chloroquine (CQ), and MG132 at the indicated concentration for another 6 h or left untreated and then infected with GCRV at an MOI of 1. At 24 hpi, the cells were lysed in radioimmunoprecipitation assay (RIPA) lysis buffer containing protease inhibitor cocktail. Total lysate was analyzed by Western blotting using anti-Vdra, anti-Vdrb, anti-VP3, anti-NS38, anti-NS80, or anti-GAPDH antibody. The protein bands were quantified using Quantity One software.

### Statistical analysis.

Statistical analysis and graphs were performed and produced using GraphPad Prism 7.0 software. Data from qRT-PCR are presented as mean and standard error of the mean (SEM). The significance of results was analyzed by Student’s *t* test (*, *P* < 0.05; **, *P* < 0.01; ns, not significant).
