# Supplementary material for: Piscine Vitamin D Receptors Vdra/Vdrb in the Absence of Vitamin D Are Utilized by Grass Carp Reovirus for Promoting Viral Replication
Source: Microbiol Spectr. 2023 Jul 19;11(4):e01287-23. doi: 10.1128/spectrum.01287-23 (PMC10433867; doi:10.1128/spectrum.01287-23)
Supplement: Supplemental file 1 — Fig. S1 and Table S1. Download spectrum.01287-23-s0001.pdf, PDF file, 0.3 MB [file spectrum.01287-23-s0001.pdf]

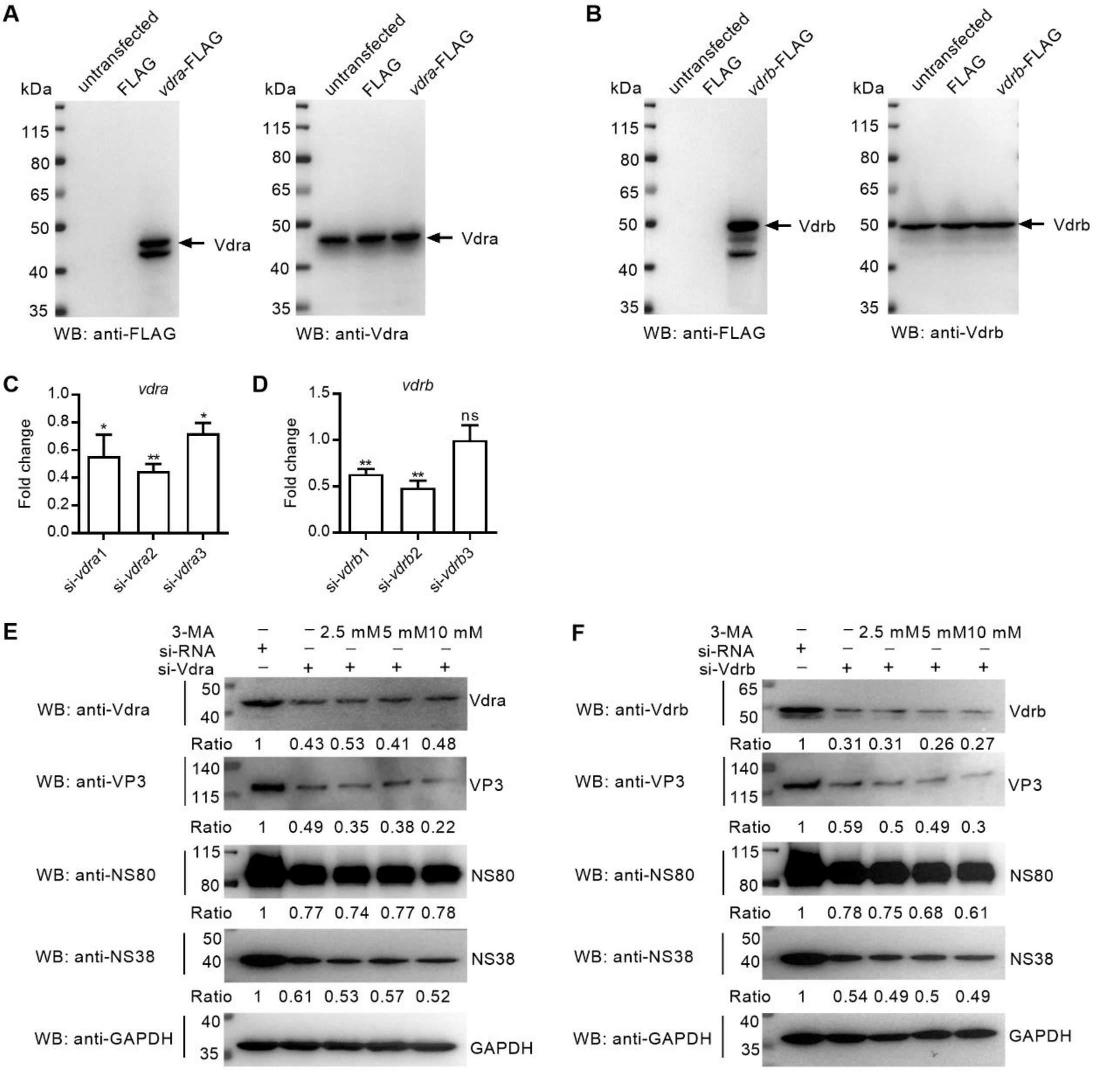

Supplemental Fig. 1. **Grass carp *vdra* and *vdrb* promote the expressions of GCRV proteins in the absence of VitD.** (**A** and **B**) The antibody specificity of grass carp Vdra or Vdrb. Specificity of grass carp Vdra or Vdrb was determined by immunoblot analysis of exogenous (left) and endogenous protein with the protein size of grass carp Vdra-FLAG or Vdrb-FLAG as positive control (right). (**C**) The effect of knockdown of grass carp *vdra* on the expression of grass carp *vdra* in CIK cells transfected with si-*vdra*1, si-*vdra*2 or si-*vdra*3. (**D**) The effect of knockdown of grass carp *vdrb* on the expression of grass carp *vdrb* in CIK cells transfected with si-*vdrb*1, si-*vdrb*2 or si-*vdrb*3. For **C** and **D**, the asterisk above the error bars indicated statistical significance using the group transfected with siRNA as the control group. **p* < 0.05, ***p* < 0.01; ns, not significant. (**E**) The effect of 3-MA on the degradation of GCRV proteins mediated by knockdown of grass carp Vdra. (**F**) The effect of 3-MA on the degradation of GCRV proteins mediated by knockdown of grass carp Vdrb. For **E** and **F**, CIK cells seeded in 6-well plates overnight were transfected with 100 nM siRNA, si-Vdra or si-Vdrb. After 24 h transfection, the cells were treated with 3-MA at the indicated concentration for another 6 h or left untreated. Then, these cells were collected and used for Western blotting. Protein bands were quantified by Image J.

Table S1. Primers used for the present study.

Primers	Sequences (5'-3')	Application
vdra-F1	CCAAGCTTATGAACCTGACAGAGACTGGG	Ligated to p3xFLAG-CMV™-14 vector
vdra-R1	GGGGTACCGAGGACACCTCGCTGCCGA	
vdrb-F1	ACAAGCTTATGGAGTCAGCCGTTAGTA	
vdrb-R1	GGGGTACCGAAGTGACCTGTCCGCC	
rxrga-F1	CCGAATTCAATGGATAATAACGACA	
rxrga-R1	GGGGTACCGTAATTTGATGCGGTG	
rxrgb-F1	CCAAGCTTATGAGCTCCATGGTGGC	
rxrgb-R1	GGGGTACCGTGATCTGATGTGGCGC	
rxrbb-F1	CCAAGCTTATGAACTCCTTACCGC	
rxrbb-R1	CGGGATCCGTGGGGCGACTCCAGC	
vdrb-F2	ACAAGCTTATGGAGTCAGCCGTTAGTA	Ligated to pTurboGFP-N vector
vdrb-R2	ACTGGTACCGAAGTGACCTGTCCGCC	
q-rig-I-F	ACTACACTGAACACCTGCGGAA	
q-*rig*-I-R	GCATCTTTAGTGCGGGCG	
q-*mda5*-F	CAGGAGCGACTCTTGGACTATG	
q-*mda5*-R	AAAGACGGTTTATTTGAATGGAAG	
q-*mavs*-F	GACCGTAAGAAGTCAGCCTCC	
q-*mavs*-R	CCTGAATAACTCTTGATAGCCCTC	
q-*tbk1*-F	CCAGGAGAAATGTTGGGGC	
q-*tbk1*-R	TGTAGATGTGGTGGAGTGTCGC	
q-*irf3*-F	ACTTCAGCAGTTTAGCATTCCC	
q-*irf3*-R	GCAGCATCGTTCTTGTTGTCA	
q-*irf7*-F	CGCCTGTGTTCGTCACTCGT	
q-*irf7*-R	GGTGGTTGGAAAGCGTATTGG	
q-*ifn1*-F	AAGCAACGAGTCTTTGAGCCT	
q-*ifn1*-R	GCGTCCTGGAAATGACACCT	
q-*mx1*-F	CTGGGGAGGAAGTAAAGTGTTCT	
q-*mx1*-R	CAGCATGGATTCTGCCTGG	
q-*rxrbb*-F	TAGATTCGCCACTGTCAGCC	
q-*rxrbb*-R	TTCCTGACTGTGCGCTTGAA	
q-*vdra*-F	GTGGACACTAAGCTGAACTTC	
q-*vdra*-R	GACGCGAGTTGTCCTCCTCG	
q-*vdrb*-F	GGAGATCGTAAGATGAATCTG	
q-*vdrb*-R	ACCGGTGCCCTCATCTTCCT	
q-*β-actin*-F	GGCTGTGCTGTCCCTGTA	
q-*β-actin*-F	GGGCATAACCCTCGTAGAT	
q-*18s*-F	ATTTCCGACACGGAGAGG	
q-*18s*-R	CATGGGTTTAGGATACGCTC	
q-*ef1α*-F	CAGCACAAACATGGGCTGGTTC	
q-*ef1α*-R	ACGGGTACAGTTCCAATACCTCCA	
q-*fdft1*-F	GGGCCAAGAGTATCAGGACG	
q-*fdft1*-R	GCAGAGAAGAGTCGGGACAG	
q-*hmgcr*-F	ACAAGCCTAACCCTGTCACG	
q-*hmgcr*-R	GGCATGTCTGGCTCAATCCT	
q-*srebf1*-F	GGAAACTCAGTTGACCCGCT	Quantitative real-time PCR

q-*srebf1*-R	TGAGTATTCCCCATCCCCGT	
q-*ldlr*-F	CTGGGCTGTGTCAATGCAAC	
q-*ldlr*-R	CCAGTGGGACACAGACACTC	
q-*lxra*-F	AGAAGAGCAGATCCGACTGA	
q-*lxra*-R	GTTGTCGAACCTCTCGGTTA	
